# Single nucleotide polymorphism discovery in cutthroat trout subspecies using genome reduction, barcoding, and 454 pyro-sequencing

**DOI:** 10.1186/1471-2164-13-724

**Published:** 2012-12-23

**Authors:** Derek D Houston, David B Elzinga, Peter J Maughan, Scott M Smith, John SK Kauwe, R Paul Evans, Ryan B Stinger, Dennis K Shiozawa

**Affiliations:** 1 Department of Biology, Brigham Young University, Provo, UT, 84602, USA; 2 Department of Plant and Wildlife Sciences, Brigham Young University, Provo, UT, 84602, USA; 3 Department of Microbiology and Molecular Biology, Brigham Young University, Provo, UT, 84602, USA; 4 Curator of Fishes, Monte L. Bean Life Science Museum, Brigham Young University, Provo, UT, 84602, USA

**Keywords:** Conservation genetics, Genetic admixture, Hybridization, KASPar, *Oncorhynchus clarkii*, *Oncorhynchus mykiss*, Population genomics, SNP, Rainbow trout

## Abstract

**Background:**

Salmonids are popular sport fishes, and as such have been subjected to widespread stocking throughout western North America. Historically, stocking was done with little regard for genetic variation among populations and has resulted in genetic mixing among species and subspecies in many areas, thus putting the genetic integrity of native salmonid populations at risk and creating a need to assess the genetic constitution of native salmonid populations. Cutthroat trout is a salmonid species with pronounced geographic structure (there are 10 extant subspecies) and a recent history of hybridization with introduced rainbow trout in many populations. Genetic admixture has also occurred among cutthroat trout subspecies in areas where introductions have brought two or more subspecies into contact. Consequently, management agencies have increased their efforts to evaluate the genetic composition of cutthroat trout populations to identify populations that remain uncompromised and manage them accordingly, but additional genetic markers are needed to do so effectively. Here we used genome reduction, MID-barcoding, and 454-pyrosequencing to discover single nucleotide polymorphisms that differentiate cutthroat trout subspecies and can be used as a rapid, cost-effective method to characterize the genetic composition of cutthroat trout populations.

**Results:**

Thirty cutthroat and six rainbow trout individuals were subjected to genome reduction and next-generation sequencing. A total of 1,499,670 reads averaging 379 base pairs in length were generated by 454-pyrosequencing, resulting in 569,060,077 total base pairs sequenced. A total of 43,558 putative SNPs were identified, and of those, 125 SNP primers were developed that successfully amplified 96 cutthroat trout and rainbow trout individuals. These SNP loci were able to differentiate most cutthroat trout subspecies using distance methods and Structure analyses.

**Conclusions:**

Genomic and bioinformatic protocols were successfully implemented to identify 125 nuclear SNPs that are capable of differentiating most subspecies of cutthroat trout from one another. The ability to use this suite of SNPs to identify individuals of unknown genetic background to subspecies can be a valuable tool for management agencies in their efforts to evaluate the genetic structure of cutthroat trout populations prior to constructing and implementing conservation plans.

## Background

Single nucleotide polymorphisms (SNPs) are powerful genetic markers that are increasingly being used in phylogenetic and population genetic studies [1,2]. Advances in high-throughput sequencing technologies have made SNP identification faster and cheaper than traditional methods that utilize Sanger sequencing (see Metzker [3] for a review of next-generation techniques). The relative ease by which SNPs can now be identified makes SNP discovery more attainable [2,4]. Indeed, many researchers have recently used next generation sequencing to detect SNPs in a variety of organisms [5-9]. SNP discovery in salmonid fishes has garnered much attention in recent years [10-19], but there is a growing need for additional SNP discovery in certain groups of salmonids.

Cutthroat trout, *Oncorhynchus clarkii*, a native western North American salmonid, has ten extant subspecies (and two extinct subspecies). The species appears to be monophyletic [20], and diversification among subspecies is postulated to have begun approximately two million years ago [20,21]. The extant subspecies are: Bonneville cutthroat trout (*O. c. utah*) in the Bonneville Basin; Coastal cutthroat trout (*O. c. clarki*) in coastal drainages from Alaska to northern California; Colorado River cutthroat trout (*O. c. pleuriticus*) in the upper Colorado River basin; Greenback cutthroat trout (*O. c. stomias*) in the Arkansas and the South Platte river basins in eastern Colorado (federally listed as threatened); Lahontan cutthroat trout (*O. c. henshawi*) in the western Lahontan Basin of Nevada and several closed basins in Oregon (federally listed as threatened); Humboldt cutthroat trout (*O. c. humboldtensis*) in the Humboldt River in the eastern portion of the Lahontan Basin recently designated as a separate sub-species from the Lahontan cutthroat trout [22]; Paiute cutthroat trout (*O. c. seleniris*) in Silver Creek on the eastern slope of the Sierra Nevada (federally listed as threatened); Rio Grande cutthroat trout (*O. c. virginalis*) in tributaries to the Rio Grande in southern Colorado and New Mexico; Westslope cutthroat trout (*O. c. lewisi*) in drainages of the Rocky Mountains in Alberta, British Columbia, northern Idaho, and Montana, with disjunct populations in Oregon and Washington; Yellowstone cutthroat trout (*O. c. bouvieri*) in the Yellowstone River, Yellowstone Lake, and the upper Snake River drainages of Wyoming, Idaho, and Montana.

The Snake River fine spotted cutthroat trout (in the upper Snake River) was designated as a separate subspecies, *O. c. behnkei*[23]. The only documented differences between it and sympatric Yellowstone cutthroat trout are the spotting pattern and behavior, whereas other morphological and meristic characters are the same [23,24]. Genetic analyses have not revealed differences between Snake River fine spotted and Yellowstone cutthroat trout [21,25,26]. Unfortunately, Montgomery [23] did not designate a type specimen when he named *O. c. behnkei*, thus technically rendering the subspecies designation invalid. Therefore, we treat the Yellowstone cutthroat trout and the Snake River fine spotted cutthroat trout as *O. c. bouvieri*. Similarly, while Paiute cutthroat trout have fewer spots than Lahontan cutthroat trout (many have no spots), they do not differ from Lahontan cutthroat trout with any other morphological or meristic characters that have been examined [24], nor do the two subspecies appear to be genetically distinct based on electrophoretic data [27]. Recently, Finger *et al.*[28] characterized six SNPs that differentiate rainbow trout from Lahontan and Paiute cutthroat trout, but the two cutthroat trout subspecies were identical at all six SNP loci. Additionally, Paiute cutthroat trout and Lahontan cutthroat trout carried identical haplotypes of the second subunit of the NADH dehydrogenase-ubiquinone oxidoreductase enzyme complex I (ND2) of the mitochondrial genome [21]. Hence, Paiute cutthroat trout and Lahontan cutthroat trout are likely to be very similar genetically.

The Bonneville cutthroat trout includes a morphologically and ecologically unique lineage of cutthroat trout in the Bear River drainage [24,29]. Based on allozyme [30,31] and mtDNA [21,26,32] data, the Bear River strain of Bonneville cutthroat trout (hereafter referred to as Bear River cutthroat trout for simplicity) are genetically more closely related to Yellowstone cutthroat trout than to the Bonneville cutthroat trout in the main Bonneville Basin [26,30-32], although an associated taxonomic revision has not yet been made. The sister relationship between Yellowstone and Bear River cutthroat trout makes biogeographic sense because the Bear River was part of the upper Snake River drainage until the late Pleistocene, at which time the Bear River was redirected into the Bonneville Basin (~35 Ka) [33-35]. Contemporary gene flow between the Bear River and other drainages within the Bonneville Basin is prevented by the Great Salt Lake. While the cutthroat trout in the Bear River system are currently classified as Bonneville cutthroat trout, herein we treat them as an additional lineage within the species *O. clarkii*.

Despite these unique lineages, cutthroat trout were stocked within and among major drainages with little concern for genetic variability among subspecies [24,36]. Introgression among cutthroat trout subspecies has resulted from these stocking practices. Additionally, rainbow trout (*Oncorhynchus mykiss*) have been stocked extensively throughout western North America. Rainbow trout readily hybridize with cutthroat trout in areas where the two species did not formerly co-occur, posing a serious threat to the genetic integrity of the native cutthroat trout populations [25,37-39]. Rainbow trout x cutthroat trout hybrids can be identified reasonably well using morphological and meristic characters, however in populations with extensive introgressive hybridization this is not always the case, nor are hybrids between cutthroat trout subspecies easily recognized. Because of widespread intra- and interspecific hybridization, management agencies have increased efforts to assess the genetic composition of native cutthroat trout populations. A range of genetic markers have been used to assess introgressive hybridization in cutthroat trout populations, and recently SNPs useful for species identification and detecting introgression between rainbow trout and cutthroat trout have been developed [28,40,41]. Some SNPs differentiate certain subspecies of cutthroat trout from rainbow trout (e.g., rainbow trout vs. westslope cutthroat trout [39,42]; rainbow trout vs. westslope and Yellowstone cutthroat trout [43]; rainbow trout vs. westslope, Yellowstone, coastal, and Lahontan cutthroat trout [44]; rainbow trout vs. westslope, Bonneville, Yellowstone, coastal and Lahontan cutthroat trout [45]). However, it remains unclear whether these SNPs are unique to those cutthroat trout subspecies, or if they are shared with other subspecies not included in those studies. Here we use next-generation sequencing technologies to identify additional nuclear SNPs that collectively characterize most of the cutthroat trout subspecies. Specifically, we used genome reduction, MID-barcoding, and 454-pyrosequencing in an attempt to discover nuclear SNPs that differentiate nine lineages of cutthroat trout (i.e., Bear River cutthroat, Bonneville cutthroat, coastal cutthroat, Colorado River cutthroat, greenback cutthroat, Lahontan cutthroat, Rio Grande cutthroat, westslope cutthroat, and Yellowstone cutthroat) and rainbow trout (including Columbia redband trout and steelhead). These SNPs were used to develop a SNP assay that can be easily used to evaluate the genetic integrity of cutthroat trout populations across the entire range of the species. The SNP assays are based on KASPar^TM^ genotyping chemistry and were detected using the Fluidigm dynamic array platform.

## Methods

A brief overview of our methods is as follows: DNA was extracted from tissue samples, followed by genome reduction and 454 pyro-sequencing. The sequences produced were assembled and scanned for SNPs bioinformatically. SNPs were then genotyped and SNP diversity analyses were performed. Individuals that yielded unexpected results according to *a priori* subspecies designations were re-sequenced for the ND2 mitochondrial DNA gene using Sanger sequencing for further evaluation.

### DNA extraction

Fin clips or muscle tissues were obtained from thirty-six individuals representing nine sub-species of cutthroat trout, plus the Bear River cutthroat trout and rainbow trout (including Columbia redband trout and steelhead, which are unique forms of *O. mykiss*) from the Monte L. Bean Life Science Museum ichthyological collection (Table 1). The samples were collected by field biologists familiar with cutthroat trout subspecies identifications based on phenotypic characters. While subspecies identifications are typically accurate when made by experts, especially when accounting for geographic distribution, the presence of cryptic hybrids among these samples is possible. We were unable to obtain tissues for Paiute cutthroat trout, but given the lack of distinguishing genetic characteristics between them and Lahontan cutthroat trout, they are likely very similar to Lahontan cutthroat trout. Whole genomic DNA was extracted using Qiagen DNeasy Tissue kits following the manufacturer’s recommended protocol. All extracted DNA was quantified using a NanoDrop 1000 Spectrophotometer (NanoDrop Technologies Inc., Montchanin, DE), and each sample was diluted to a concentration of 150 ng/μl using nuclease free water.

**Table 1:** Sampling information

**Species/subspecies**	**n**	**BYU #**	**Location**	**Drainage (Basin)**	**Latitude/Longitude**
Cutthroat Trout (*Oncorhynchus clarkii*)					
Bonneville cutthroat trout	10	**33807**	**Chalk Creek, UT**	**Weber River (Bonneville)**	**40° 59’ 44” N, 111° 03’ 31” W**
*O. c. utah*		**134304**	**Trout Creek, UT**	**Deep Creek Mts. (Bonneville)**	**39° 45’ 55” N, 113° 55’ 45” W**
		**135225**	**Birch Creek, UT**	**Beaver River (Bonneville)**	**38° 12’ 59” N, 112° 31’ 49” W**
		**179753**	**Nebo Creek, UT**	**Spanish Fork River (Bonneville)**	**39° 51’ 04” N, 111° 38’ 26” W**
		93975	Main Creek, UT	Provo River (Bonneville)	40**°** 19’ 02” N, 111° 20’ 06” W
		135133	Willard Creek, NV	Spring Valley	39**°** 01’ 22” N, 114° 23’ 33” W
		134577	Lost Creek, UT	Sevier River (Bonneville)	38**°** 43’ 14” N, 111° 42’ 06” W
		181736	Deadman Creek, NV	Smith Creek (Bonneville)	39**°** 19’ 40” N, 114° 08’ 23” W
		181774	Pine Creek, NV	Spring Valley	38**°** 59’ 09” N, 114° 22’ 34” W
		181752	Hampton Creek, NV	Hampton Creek (Bonneville)	39**°** 14’ 39” N, 114° 06’ 28” W
Bear River Bonneville cutthroat trout	10	**135526**	**Yellow Creek, UT**	**Bear River**	**40° 58’ 58” N, 110° 57’ 35” W**
*O. c. utah*		**136684**	**Dog Creek, WY**	**Bear River**	**43° 37’ 52” N, 110° 48’ 11” W**
		**136733**	**Cottonwood Creek, WY**	**Upper Salt River (Bear R.)**	**42° 37’ 52” N, 110° 50’ 34” W**
		**179631**	**Smith’s Fork, WY**	**Bear River**	**42° 10’ 34” N, 110° 53’ 22” W**
		135528	Yellow Creek, UT	Bear River	40° 58’ 58” N, 110° 57’ 35” W
		179602	Daniel Fish Hatchery, WY	N/A	
		179682	Raymond Creek, WY	Bear River (Bonneville)	42° 16’ 32” N, 111° 00’ 33” W
		179702	Woodruff Reservoir, UT	Bear River (Bonneville)	41° 27’ 52” N, 111° 19’ 27” W
		179703	Woodruff Reservoir, UT	Bear River (Bonneville)	41° 27’ 52” N, 111° 19’ 27” W
		239318	Weber River, UT	Bonneville Basin	40° 46’ 54” N, 110° 59’ 41” W
Coastal cutthroat trout	10	**91991**	**Slippery Lake, AK**	**Kuiu Island (Pacific Ocean)**	**56° 13’ N, 134° 13’ W**
*O. c. clarki*		**92742**	**Abernathy Creek, OR**	**Willamette River (Columbia R.)**	**45° 19’ 56” N, 122° 32’ 32” W**
		130929	Lost Creek, OR	Coos River (Pacific Ocean)	43° 09’ 48” N, 123° 42’ 45” W
		130905	Hemlock Creek, WA	Cowlitz River (Columbia R.)	46° 15’ 48” N, 122° 43’ 45” W
		130906	Hemlock Creek, WA	Cowlitz River (Columbia R.)	46° 15’ 48” N, 122° 43’ 45” W
		91953	Hackleman Creek, OR	Willamette River (Columbia R.)	44° 24’ 45” N, 122° 02’ 32” W
		90938	Margaret Lake, AK	Revillagigedo Island (Pacific)	55° 41’ 14” N, 131° 36’ 24” W
		90946	Margaret Lake, AK	Revillagigedo Island (Pacific)	55° 41’ 14” N, 131° 36’ 24” W
		91504	Flynn Creek, OR	Coastal Range (Pacific)	44° 31’ 30” N, 123° 51’ 44” W
		91505	Flynn Creek, OR	Coastal Range (Pacific)	44° 31’ 30” N, 123° 51’ 44” W
Colorado River cutthroat trout	10	**94736**	**Little Snake River, CO**	**Duchesne River (Colorado R.)**	**40° 59’ 15” N, 107° 58’ 08” W**
*O. c. pleuriticus*		**130805**	**Muddy Creek, WY**	**Blacks Fork River (Colorado R.)**	**41° 10’ 20’ N, 110° 39’ 12” W**
		**134410**	**North Elk Creek, CO**	**White River (Colorado R.)**	**39° 54’ 54” N, 107° 40’ 18” W**
		**134387**	**Avintaquin Creek, UT**	**Yampa River (Colorado R.)**	**40° 07’ 26” N, 110° 44’ 25” W**
		135182	White River, UT	Price River (Colorado R.)	39° 55’ 26” N, 111° 03’ 07” W
		134518	Cutthroat Creek, CO	Colorado River	37° 06’ 18” N, 106° 41’ 34” W
		134537	Navajo River, CO	San Juan River (Colorado R.)	37° 11’ 55” N, 106° 39’ 49” W
		134658	Steelman Creek, CO	Williams Fork (Colorado R.)	39° 45’ 24” N, 105° 55’ 57” W
		134688	Cabin Creek, CO	Ranch Creek (Colorado R.)	39° 58’ 25” N, 105° 44’ 30” W
		134748	Fryingpan River, CO	Roaring Fork River (Colorado R.)	39° 22’ 01” N, 107° 01’ 58” W
Greenback cutthroat trout	10	**131909**	**Como Creek, CO**	**Arkansas River (Missouri R.)**	**38° 32’ 02” N, 106° 14’ 17” W**
*O. c. stomias*		**132038**	**South Prong Hayden Creek, CO**	**Arkansas River (Missouri R.)**	**38° 18’ 47” N, 105° 49’ 25” W**
		**179073**	**Lower Bear Creek, CO**	**Arkansas River (Missouri R.)**	**38° 48’ 33” N, 104° 54’ 18” W**
		**180022**	**Beaver Creek, UT**	**Colorado River**	**38° 21’ 17” N, 109° 15’ 48” W**
		179085	North Taylor Creek, CO	Arkansas River (Missouri R.)	38° 07’ 14” N, 105° 36’ 15” W
		142903	Coon Creek, CO	Plateau Creek (Colorado R.)	39° 05’ 49” N, 108° 07’ 16” W
		134692	Cabin Creek, CO	Ranch Creek (Colorado R.)	39° 58’ 25” N, 105° 44’ 30” W
		179022	Severy Creek, CO	Arkansas River (Missouri R.)	38° 52’ 33” N, 105° 02’ 53” W
		78265	West Antelope Creek, CO	Gunnison River (Colorado R.)	38° 35’ 12” N, 106° 59’ 59” W
		187526	Carr Creek, CO	Colorado River	39° 35’ 09” N, 108° 32’ 03” W
Lahontan Basin cutthroat trout	10	**96518**	Doudy Pond, UT*	**(Pyramid Lake) (Lahontan)**	**41° 01’ 17” N, 113° 58’ 12” W**
*O. c. henshawi*		96517	Doudy Pond, UT*	(Pyramid Lake) (Lahontan)	41° 01’ 17” N, 113° 58’ 12” W
*O. c. humboldtensis*		**90414**	**Coyote Creek, NV**	**Humboldt River (Lahontan)**	**40° 58’ 42” N, 116° 12’ 18” W**
		182066	Baker Lake, NV	Baker Creek (Bonneville)	38° 57’ 27” N, 114° 18’ 31” W
		95362	Virgin Creek, NV	Alvord Basin	41° 37’ 36” N, 119° 09’ 15” W
		97748	Segunda Creek, NV	Humboldt River (Lahontan)	40° 31’ 59” N, 115° 29’ 10” W
		90741	McDermitt Creek, OR	Quinn River (Lahontan)	42° 00’ 20” N, 118° 00’ 55” W
		91109	Indian Creek, NV	Humboldt River (Lahontan)	40° 19’ 37” N, 116° 43’ 42” W
		90414	Coyote Creek, NV	Humboldt River (Lahontan)	41° 19’ 42” N, 116° 33’ 02” W
		91162	Mahogany Creek, NV	Summit Lake	41° 31’ 01” N, 118° 57’ 24” W
Rio Grande cutthroat trout	10	**57036**	**Torcido Creek, CO**	**Rio Grande**	**37° 45’ 51” N, 105° 21’ 35” W**
*O. c. virginalis*		**90712**	**Peralta Creek, NM**	**Rio Grande**	**35° 41’ 33” N, 106° 28’ 02” W**
		**99839**	**Little Vermejo, NM**	**Canadian River (Mississippi R.)**	**36° 59’ 18” N, 105° 08’ 04” W**
		**99858**	**Middle Fork Carnero Creek, CO**	**Rio Grande**	**37° 58’ 55” N, 106° 25’ 32” W**
		90714	Peralta Creek, NM	Rio Grande	35° 41’ 33” N, 106° 28’ 02” W
		99862	Middle Fork Carnero Creek, CO	Rio Grande	37° 58’ 55” N, 106° 25’ 32” W
		99844	Little Vermejo, NM	Canadian River (Mississippi R.)	36° 59’ 18” N, 105° 08’ 04” W
		57045	Ventero Creek, NM	Rio Grande	36° 59’ 49” N, 105° 27’ 58” W
		99841	Little Vermejo, NM	Canadian River (Mississippi R.)	36° 59’ 18” N, 105° 08’ 04” W
		90732	Peralta Creek, NM	Rio Grande	35° 41’ 33” N, 106° 28’ 02” W
Westslope cutthroat trout	10	**239020**	**Black Bear, MT**	**South Fork Flathead River**	**47° 44’ 41” N, 113° 23’ 00” W**
*O. c. lewisi*		**239024**	**White Gulch, MT**	**Missouri River**	**46° 37’ 10” N, 111° 28’ 51” W**
		**187969**	**Bull River, BC**	**Kootenay River (Columbia R.)**	**49° 33’ 58” N, 115° 19’ 26” W**
		**181526**	**John Day River, OR**	**Columbia River**	**44° 16’ 57” N, 118° 32’ 43” W**
		239021	Black Bear, MT	South Fork Flathead River	47° 44’ 41” N, 113° 23’ 00” W
		239022	McGuire, MT	Kootenai River	48° 41’ 20” N, 115° 16’ 22“ W
		239023	McGuire, MT	Kootenai River	48° 41’ 20” N, 115° 16’ 22“ W
		239025	White Gulch, MT	Missouri River	46° 37’ 10” N, 111° 28’ 51” W
		244201	Crazy Creek, BC	South Thompson River	51° 01’ 43” N, 124° 33’ 31” W
		244202	Bow River, BC	South Saskatchewan River	51° 36’ 37” N, 116° 07’ 56” W
Yellowstone cutthroat trout	10	**133863**	**East Tensleep Creek, WY**	**Bighorn River (Missouri R.)**	**44° 12’ 11” N, 107° 09’ 53” W**
*O. c. bouvieri*		**136780**	**Glade Creek, WY**	**Upper Snake River (Columbia)**	**44° 05’ 40” N, 110° 43’ 59” W**
		**136834**	**Willow Creek, WY**	**Lower Salt River**	**42° 50’ 45” N, 110° 52’ 54” W**
		136668	North Fork Horse Creek, WY	Upper Snake River (Columbia)	43° 22’ 51” N, 110° 37’ 43” W
		141843	Forest Creek, WY	Upper Snake River (Columbia)	44° 10’ 14” N, 110° 35’ 49” W
		141946	Sickle Creek, WY	Upper Snake River (Columbia)	44° 11’ 28” N, 110° 23’ 05” W
		142366	Elk Antler Creek, WY	Yellowstone River (Missouri R.)	44° 36’ 01” N, 110° 28’ 39” W
		90212	Hatchery Creek, WY	Yellowstone River (Missouri R.)	44° 32’ 57” N, 110° 24’ 03” W
		179112	Mill Creek, WY	Yellowstone River (Missouri R.)	44° 26’ 59” N, 107° 27’ 09” W
		179114	Mill Creek, WY	Yellowstone River (Missouri R.)	44° 26’ 59” N, 107° 27’ 09” W
Rainbow Trout (*Oncorhynchus mykiss*)					
Rainbow Trout	1	**187968**	**Rio Fuerte, Mexico**	**Gulf of California**	**Unknown**
Columbia Redband Trout	3	**142374**	**McCoy Creek, OR**	**Harney-Malheur Basin**	**42° 58’ 33” N, 118° 42’ 32” W**
		**98251**	**West Fork Bruneau River, NV**	**Snake River (Columbia R.)**	**41° 56’ 02” N, 115° 40’ 29” W**
		**94217**	**West Fork Jarbridge River, NV**	**Snake River (Columbia R.)**	**41° 48’ 57” N, 115° 24’ 38” W**
Steelhead	2	**93748**	**Kootenay River, BC**	**Columbia River**	**49° 19’ N, 117° 39’ W**
		97060	Midway Hatchery, UT	N/A	

Cutthroat trout individuals used in this study are listed along with sampling locality information for each. Individuals that were included on the 454 plate are highlighted with bold font. All individuals were used to validate/verify SNPs using Fluidigm nanofluidic chip arrays.

*Transplanted population – source population was Morrison Creek, NV, which is believed to be a Pyramid Lake strain of Lahontan cutthroat.

Some of the DNA samples that were extracted were not high enough quality to be included in the genomic reduction and 454-pyrosequencing steps (outlined below). In most cases this resulted in simply using one less individual from a subspecies, but was more problematic for Lahontan and Humboldt cutthroat trout where only one individual of each subspecies had DNA of a high enough quality to include. To ensure getting enough reads, those two individuals were pooled, resulting in a Lahontan Basin complex where Lahontan and Humboldt cutthroat were treated as a single unit. Hereafter we refer to both groups as Lahontan Basin cutthroat, but this is merely for convenience, and is not meant to be a statement regarding a taxonomic update.

To verify that the discovered SNPs were able to differentiate subspecies of cutthroat trout from each other and from rainbow trout, DNA was extracted from an additional sixty cutthroat trout individuals representing ten trout lineages. These sixty samples, along with the thirty-six samples that were extracted for 454-pyrosequencing, were used to verify SNPs. Hence, each cutthroat trout lineage was represented by ten individuals, and rainbow trout was represented by six individuals for SNP verification. In an attempt to account for geographic variation within cutthroat trout subspecies, samples were included from populations different from those included on the initial SNP discovery panel when possible (Table 1). Samples were chosen from populations that are believed to be non-admixed, but this was not verifiable in all cases.

### Genomic reduction and 454-pyrosequencing

We followed the genomic reduction methodology described in detail by Maughan *et al*. [6]. In brief, genomic reduction was carried out using restriction enzymes *Eco*RI and *Bfa*I to double digest genomic DNA at restriction sites that were conserved across all sub-species, and then attaching *Eco*RI and *Bfa*I adapter sequences to the sticky ends using T4 DNA ligase. Approximately 90% of the genome was then discarded through size exclusion via spin chromatography and biotin-streptavidin paramagnetic bead separation. Prior to 454-pyrosequencing, unique MID-barcodes were added to the remaining restriction fragments for each individual sample via polymerase chain reaction (PCR). DNA concentrations of the final PCR products were quantified using picogreen fluorescent dye, and then all PCR products were pooled in equimolar amounts to obtain a single sample totaling 5 μg of DNA. Gel electrophoresis was performed on the pooled sample in a single lane of a 1.5% agarose gel, and DNA fragments ranging from ~450-600 base pairs (bp) in size were removed and then used for 454-pyrosequencing. Sequencing was performed with Titanium reagents without DNA fragmentation on a 454 Life Sciences Genome Sequencer FLX located in the DNA Sequencing Center at Brigham Young University.

### Assembly and SNP discovery

Following 454-pyrosequencing, CLCBio Workbench bioinformatic software v.3.6.1 (Katrinebjerg, Aarhus N, Denmark) was used to separate the DNA sequences according to the MID-barcodes that were attached to DNA fragments from each of the individual samples. All reads from all subspecies were pooled and a *de novo* assembly was created using Newbler v.2.6 (454 Life Sciences 2006–2011), after which all reads from all subspecies were mapped onto this assembly using the reference mapping function in CLCBio Workbench.

Putative autapomorphic SNPs were identified by comparing sequences derived from a single cutthroat trout subspecies to the sequences of the other subspecies combined. Comparisons were completed using SNP_Finder_Plus, a custom perl script described by Maughan *et al.*[6]. A similar comparison between rainbow trout and cutthroat trout (all subspecies sequences pooled) was used to identify species specific SNPs. A subset of SNPs was selected for validation from the pool of all putative SNPs using the following criteria: 1) Polymorphisms were unique to a single subspecies of cutthroat or rainbow trout so that only potentially diagnostic SNPs were selected. 2) All reads at the SNP locus had a minimum read coverage depth ≥ 8 to exclude putative SNPs that were based on too few reads. 3) A minimum of 50 bp existed on either side of the putative SNP for primer binding sites, with no indels or ambiguities within 20 bp of the SNP. 4) The minor allele of the SNP had to consist of a minimum of 3 reads comprising at least 4% of the total alleles observed at that position or it was not considered as a SNP. This was an arbitrary cutoff designed as an extra filter to ensure that the minor allele is not erroneous (particularly for contigs with high coverage depth). 5) The SNP had at least 95% identity within the subspecies so that only alleles that were fixed or nearly fixed for a given subspecies were considered, and alleles that were unique to a small proportion of individuals within a subspecies were not. This criterion also guards against calling SNPs in misaligned sites. 6) The SNP did not appear in known repeat regions or reside within the mtDNA genome, which was determined using RepeatMasker v.3.3.0 [46] against the *Danio* reference database and by performing BLAST searches on all contigs with a rainbow trout mtDNA reference genome [GenBank: NC_001717]. We acknowledge that mtDNA SNPs are useful in many cases, but the purpose of this study was to discover unlinked nuclear markers for additional statistical power in future population genetic studies. 7) SNPs were limited to one per contig in an attempt to minimize the number of linked alleles included. Limiting the SNPs to one per contig also served as a screen to eliminate paralogous sequence variants (PSVs). A position weighted window filter was also used to determine if SNPs were in a poor alignment region. Every polymorphism that occurred within the window was assigned a score based on its relative position to the SNP, the total score was added up and if a threshold score ≥6 was reached the SNP was considered erroneous and skipped. This window filter was designed to avoid calling SNPs in misaligned regions, as well as to avoid areas that aligned paralogous loci, thus serving as an additional screen for PSVs. Primers for 288 SNP loci that met the above criteria (enough to fill three Fluidigm chips) were designed using the default settings in the PrimerPicker software program [47]. Primer sequences are listed in Additional file 1.

### SNP genotyping

Prior to genotyping the SNPs, specific target amplification (STA) was used to pre-amplify each SNP locus. STA primers are non-allele specific and do not carry a polymorphic base on their 3’ end (Additional file 1). STA reactions consisted of 2.5 μl of Qiagen 2X Multiplex PCR Master Mix, 0.5 μl of the 10X STA primer assays (which consisted of 192 μl STA primer, 192 μl constant primer [2 μl each primer per reaction × 96 reactions], and 16 μl TE buffer [10 mM Tris, 1 mM EDTA, pH 7.5]), and 0.75 μl DNase free water per reaction. A total of 3.75 μl of the STA pre-mix was combined with 1.25 μl of genomic DNA and amplified with the following thermal profile: 95°C for 15 minutes followed by 14 cycles of 95°C for 15 seconds and 60°C for 4 minutes. Following PCR, we diluted the STA products (1:100) with nuclease free water.

The SNPs were genotyped using KASPar^TM^ genotyping chemistry (KBioscience Ltd., Hoddesdon, UK) using the Fluidigm (Fluidigm Corp., South San Francisco, CA) nanofluidic 96.96 dynamic array^TM^[48], following the methods described by Maughan *et al*. [49]. End-point fluorescent images of the Fluidigm chips were obtained on an EP-1 imager (Fluidigm Corp., South San Francisco, CA). The data were analyzed using Fluidigm SNP genotyping analysis software [50].

### SNP diversity data analysis

To visualize the ability of the SNPs to discern among cutthroat trout lineages, three analyses were performed. First, a NeighborNet phylogenetic network was created using the software program SplitsTree v.4.12.6 [51]. Second, principal coordinates analysis (PCoA) was performed using the Ecodist package v. 1.2.7 [52] available in the statistical software program R v. 2.14.1 [53]. Third, the population genetic software program Structure v.2.2.3 [54] and Structure Harvester [55] were used to infer the number of distinct populations within our panel. Structure analysis was evaluated 30 times for each K (with K ranging from 2 to 18), with 1,000,000 repetitions per run after discarding an initial 100,000 repetitions as burn-in. In three instances, Structure was unable to differentiate among some subspecies (see Results). Each of those three groups of subspecies were extracted from the original input file and re-evaluated 20 times for each K (with K ranging from 1 to 6) following the same procedure as outlined above.

### MtDNA Re-sequencing

The mitochondrial ND2 gene was re-sequenced for some individual samples that did not group with their pre-defined subspecies as expected (see Results). Amplification of ND2 was achieved using PCR primers Gln56F (5’-ACT ACA CCA CTT TCT AGT AAG GTC AGC-3’) and Ala13R (5’-GCA TTC AGA AGA TGT GGG ATA AAG TC-3’). Reaction cocktails were 12.5 μl in volume, and contained ~100 ng genomic DNA, 2.25 μl nuclease free water, 0.5 μl each primer, and 6.25 μl Promega GoTaq® Hot Start Green Master Mix. The thermal profile contained an initial denature of 95°C for two minutes to activate the enzyme, 35 cycles of 95°C for 30 seconds, 48°C for 30 seconds, and 72°C for 90 seconds, followed by a rapid cool down to 12°C. The light and heavy strands were each sequenced in 10.5 μl reactions using the same primers that amplified the ND2 gene and Big Dye chemistry. Excess dye terminator was removed using Sephadex columns. Sequencing was performed on an ABI 3730xl automated sequencer located in the DNA Sequencing Center at Brigham Young University.

## Results

### 454-Pyrosequencing, genome assembly and SNP discovery

**Figure 1:**
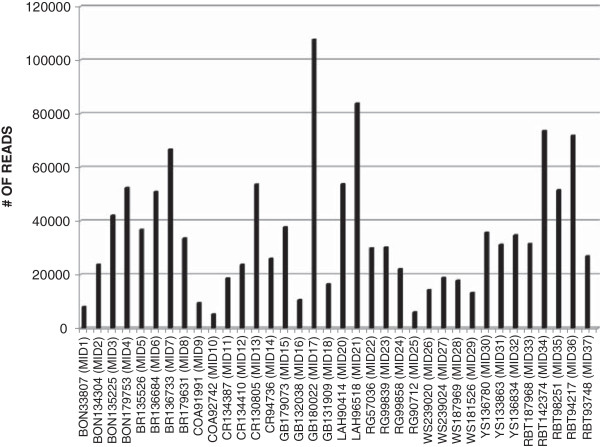
**Individual read numbers.** Number of reads for each cutthroat trout and rainbow trout individual, identified by BYU # and MID-barcode, BYU #s are prefaced with a corresponding subspecies abbreviation as follows: BR=Bear River cutthroat, BON=Bonneville cutthroat, COA=Coastal cutthroat, CR=Colorado River cutthroat, GB=Greenback cutthroat, LAH=Lahontan Basin cutthroat, RG=Rio Grande cutthroat, WS=westslope cutthroat, YS=Yellowstone cutthroat, and RBT=rainbow trout.

**Figure 2:**
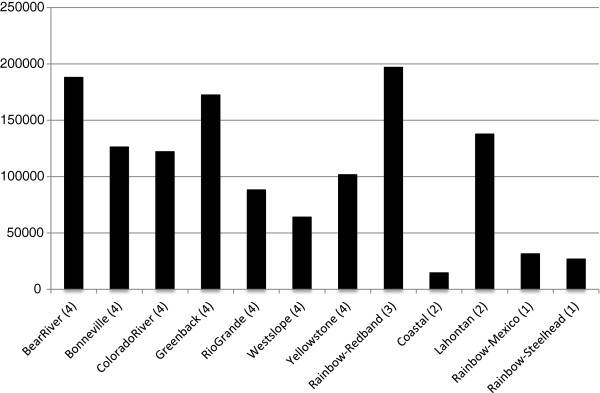
**Read numbers by subspecies.** Pooled number of reads per lineage of cutthroat trout and rainbow trout.

454-pyrosequencing produced 1,499,670 reads, with an average read length of 379 bp, for a total of 569,060,777 bp from a single run. The reads have been made publicly available via the NCBI Sequence Read Archive (Study #SRA062178). Reads were not equal for each subspecies. This inequality was likely influenced by the differences in sample size for each subspecies since N ranged from 2 to 4 for each subspecies of cutthroat that was included on the 454 plate (Table 1). Additionally, some MID-barcodes produced more reads than others (Figure 1) even though an attempt was made to mix the samples in equimolar amounts before sequencing. The discrepancy is likely due to difficulties associated with fluorometric quantification of the PCR samples before pooling and/or inaccurate pipetting during the pooling process. Of the cutthroat trout lineages, Bear River cutthroat trout had the most reads (187,976) and coastal cutthroat trout had the fewest (14,607) (see Figure 2).

We discovered 43,558 putative SNPs, of which 6383 (15%) had BLAST hits to the GenBank refseq_protein database, and 37,175 (85%) did not. Our selection process revealed that 28,887 of those putative SNPs met the first five criteria outlined above for designing SNP primers (unique to a single subspecies, ≥8X coverage at the SNP, 50 bp flanking the SNPs for primer binding sites, minor allele frequency of at least 3 reads, and 95% identical for a subspecies). Pairwise comparisons showing the number of putative SNPs between each subspecies are shown in Table 2. A total of 8,627 of these putative SNPs that met the first five criteria were excluded from primer picking because they were determined to occur in repeat regions or in the mitochondrial genome. Of the remaining 20,260 putative SNPs, we developed primers for 288 SNP loci, taking care not to choose primers from the same contig when possible in an attempt to avoid picking linked loci.

**Table 2:** Numbers of putative SNPs

	**Bear River**	**Bonneville**	**Coastal**	**Colorado River**	**Greenback**	**Lahontan Basin**	**Rio Grande**	**Westslope**	**Yellowstone**
Bear River	–	2279	482	2170	2430	5459	1807	3600	941
Bonneville	280	–	393	1182	1368	3528	1118	2268	1476
Coastal	50	63	–	314	479	407	242	228	302
Colorado River	270	148	35	–	1127	2775	969	1679	1225
Greenback	294	131	94	99	–	3604	1125	2578	1603
Lahontan Basin	854	428	74	358	499	–	2258	2722	3277
Rio Grande	273	91	24	64	63	235	–	1401	1141
Westslope	539	243	50	274	227	301	152	–	1930
Yellowstone	110	129	56	110	197	419	130	200	–

Pairwise comparisons of putative SNP loci among cutthroat trout subspecies discovered by 454 pyro-sequencing, Comparisons are shown for 6X coverage (above diagonal), and for 10X coverage (below diagonal).

### SNP genotyping and diversity analysis

A total of 125 of the subset of 288 SNP loci for which we developed primers (43%) produced clean amplification signal, yielding genotypic clusters that were visibly separated from each other and could be scored following PCR amplification and SNP genotyping. Sequence information for all 125 validated SNPs has been deposited in the GenBank database [GenBank accession #s are listed in Additional file 1]. All 125 validated SNPs are listed with the minor allele frequencies and the subspecies that carried the minor allele in Additional file 2. Westslope cutthroat trout had the lowest number of polymorphic SNPs with 20, whereas greenback cutthroat trout had the highest number of polymorphic SNPs with 75 (Table 3). Westslope cutthroat trout also had the lowest number of highly polymorphic SNPs with 7, and Bear River cutthroat had the highest number of highly polymorphic SNPs with 22 (Table 3).

**Table 3:** SNP genotyping and diversity results

	**Bear River**	**Bonneville**	**Coastal**	**Colorado River**	**Greenback**	**Lahontan Basin**	**Rio Grande**	**Westslope**	**Yellowstone**	**Rainbow**
Sample size	10	10	10	10	10	10	10	10	10	5
Polymorphic SNPs	67	55	41	65	75	45	57	20	40	19
MAF range	0.05 – 0.50	0.05 – 0.50	0.05 – 0.50	0.05 – 0.50	0.05 – 0.50	0.05 – 0.50	0.05 – 0.50	0.05 – 0.50	0.05 – 0.50	0.10 – 0.50
Average MAF	0.125	0.080	0.074	0.130	0.116	0.071	0.099	0.045	0.091	0.048
Highly polymorphic SNPs	22	11	11	20	17	11	14	7	19	10

Summary of the results from the single nucleotide polymorphism (SNP) genotyping and diversity analysis Listed are sample size, total number of polymorphic SNP loci, range of minor-allele frequencies (MAF), average MAF, and total highly polymorphic SNP loci.

The diversity panel was comprised of 95 individuals representing nine lineages of cutthroat trout (n=10 per lineage), rainbow trout (n=5), and a negative control. The total number of polymorphic SNPs ranged from 19 to 75 for these cutthroat trout subspecies and rainbow trout (Table 3). Minor-allele frequencies (MAF) ranged from 0.05 to 0.50 for each subspecies of cutthroat trout, and from 0.10 to 0.50 for rainbow trout (Table 3). The mean MAF value for all subspecies of cutthroat trout was 0.198 per SNP locus. Because SNPs are biallelic markers, the maximum MAF for any given SNP locus is 0.50, which occurs when both SNP alleles are present at equal frequencies in the sample population. Therefore, considering SNP loci that exhibited a MAF ≥ 0.3 to be highly polymorphic, the number of highly polymorphic SNPs in these cutthroat trout subspecies ranged from 7 to 22 (Table 3). Minor allele frequencies for all 125 SNPs reported here are listed in Additional file 2.

**Figure 3:**
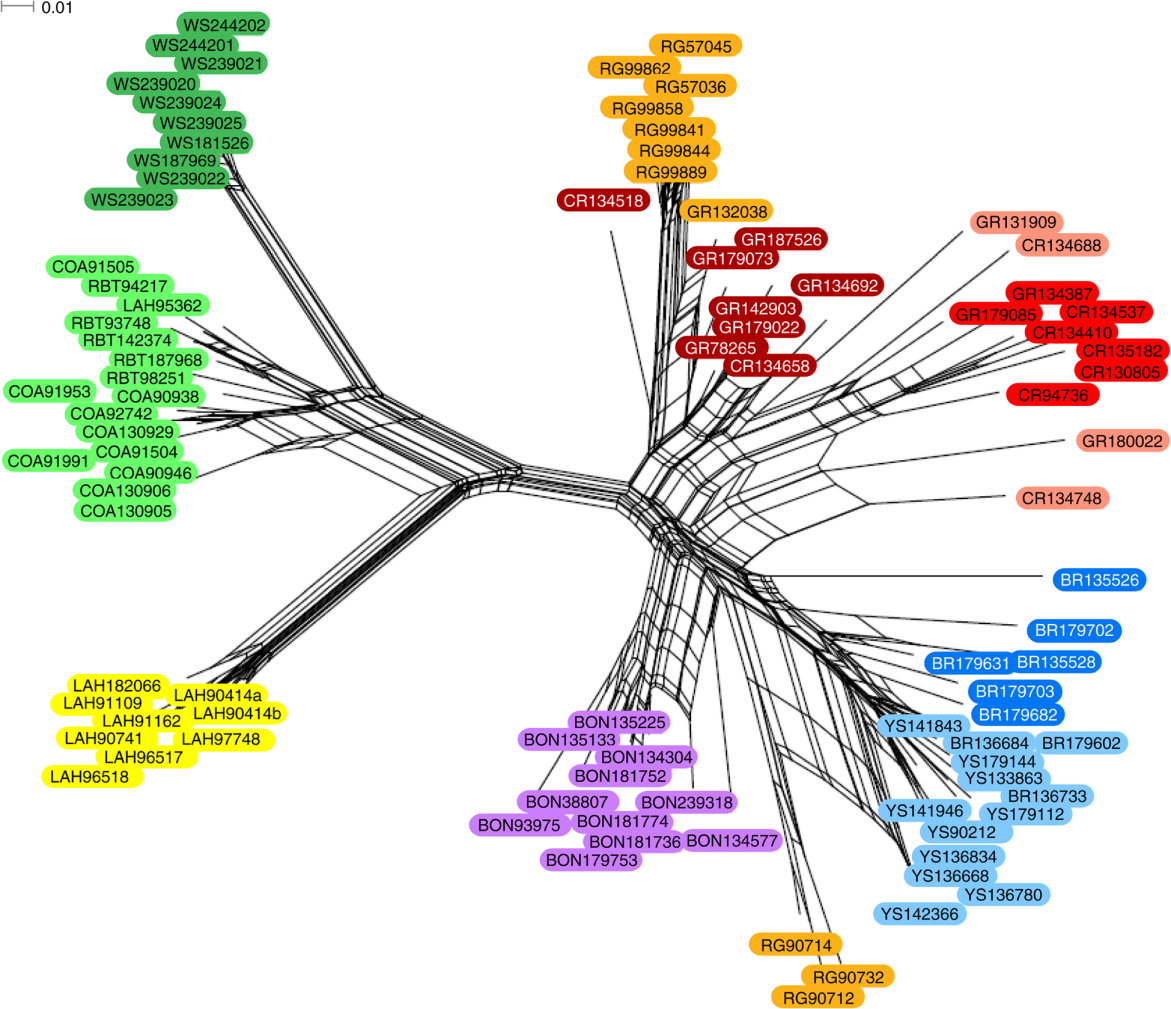
**Phylogenetic network.** NeighborNet phylogenetic network illustrating genetic distances among cutthroat trout species and rainbow trout, Individual names are represented by abbreviations for species/subspecies designations followed by BYU ID numbers. Subspecies abbreviations are as follows: BR=Bear River cutthroat, BON=Bonneville cutthroat, COA=Coastal cutthroat, CR=Colorado River cutthroat, GB=Greenback cutthroat, LAH=Lahontan Basin cutthroat, RG=Rio Grande cutthroat, WS=westslope cutthroat, YS=Yellowstone cutthroat, and RBT=rainbow trout. Individuals are highlighted with the same colors used to designate unique groups in the Structure results (see Figures 5 and 6).

The SplitsTree analysis produced a NeighborNet phylogenetic network that separates the cutthroat trout subspecies (Figure 3). Coastal cutthroat trout clustered together with rainbow trout. Westslope cutthroat clustered with the coastal-rainbow group, but with a large genetic distance between them. Lahontan Basin cutthroat trout exhibited great genetic distance between them and the other subspecies, as did the coastal-westslope-rainbow group. Of the interior cutthroat trout subspecies (i.e., Bear River, Bonneville, Colorado River, Greenback, Rio Grande and Yellowstone), Bonneville and Rio Grande cutthroat separated from the others, although Rio Grande cutthroat appear in two separate parts of the network (Figure 3). Bear River and Yellowstone cutthroat cluster together, as do Colorado River and greenback cutthroat trout. Moreover, 6 of the 95 individuals did not separate as expected according to their *a priori* subspecies designation. One Lahontan Basin cutthroat individual (LAH95362) grouped with rainbow trout rather than with the other Lahontan Basin individuals. Three Rio Grande cutthroat individuals (RG90712, RG90714, and RG90732) clustered with the group containing Yellowstone and Bear River cutthroat rather than with the other seven Rio Grande cutthroat individuals. One Bear River cutthroat individual (BR239318) grouped with Bonneville cutthroat, and one Colorado River cutthroat individual (CR134518) connected to the base of a branch leading to a group containing seven Rio Grande cutthroat individuals. Re-sequencing of the mitochondrial gene ND2 showed that the “problematic” Lahontan Basin cutthroat individual carried rainbow trout mtDNA, and that the Colorado River cutthroat individual carried Greenback cutthroat trout mtDNA. The Rio Grande and Bear River cutthroat individuals carried mtDNA sequences of their respective subspecies.

**Figure 4:**
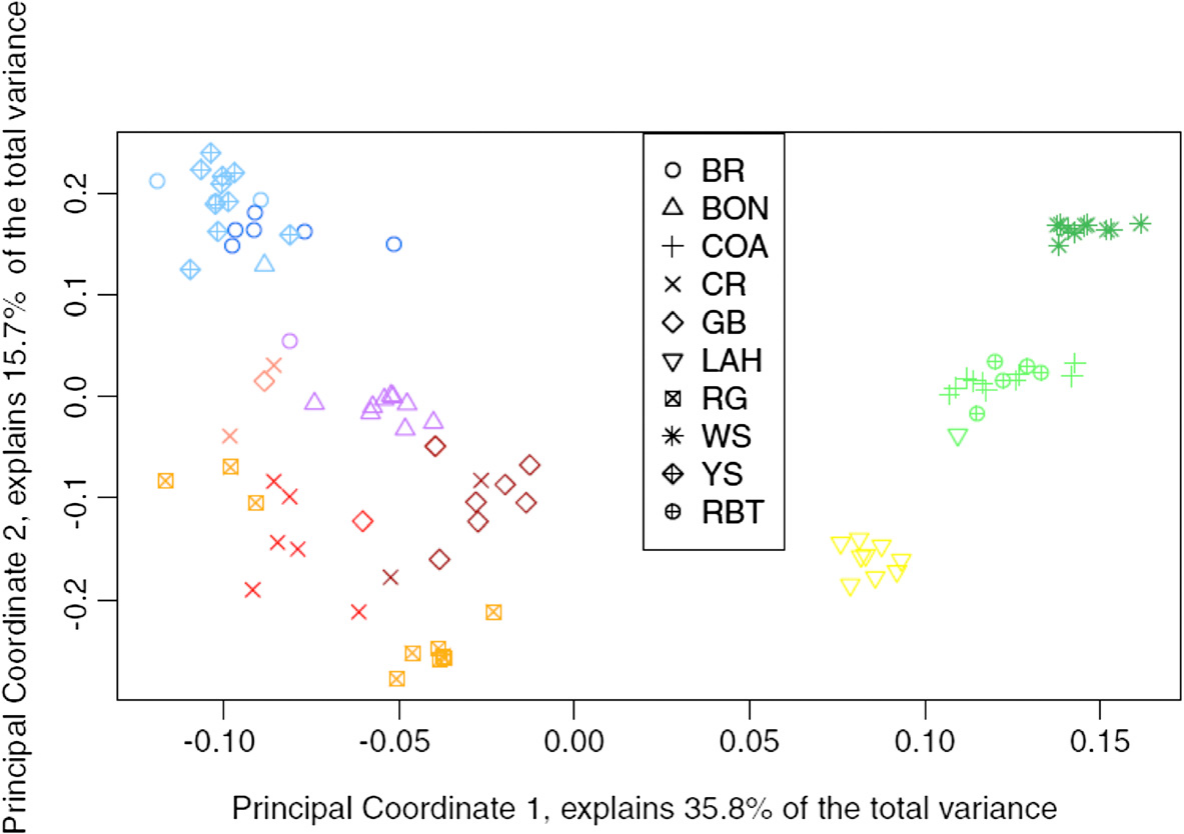
**Principal coordinates analysis plot.** Results of PCoA showing the first two principal coordinates that, combined, explain 51.5% of the observed genetic variation. Shapes on the figure legend correspond to the *a priori* subspecies designations. Subspecies are abbreviated the same as in Figures 1 and 3. Colors correspond to the population assignments made by Structure analyses (see Figures 5 and 6).

Principal coordinates analysis results showed that Principal Coordinate 1 explained 35.8% of the total variance, and Principal Coordinate 2 explained 15.7% (Figure 4). Thus, the first two principal coordinates combined explained 51.5% of the total variance observed in the distance matrix. Groups on the PCoA plot (Figure 4) replicate the groups observed on the phylogenetic network (Figure 3).

**Figure 5:**
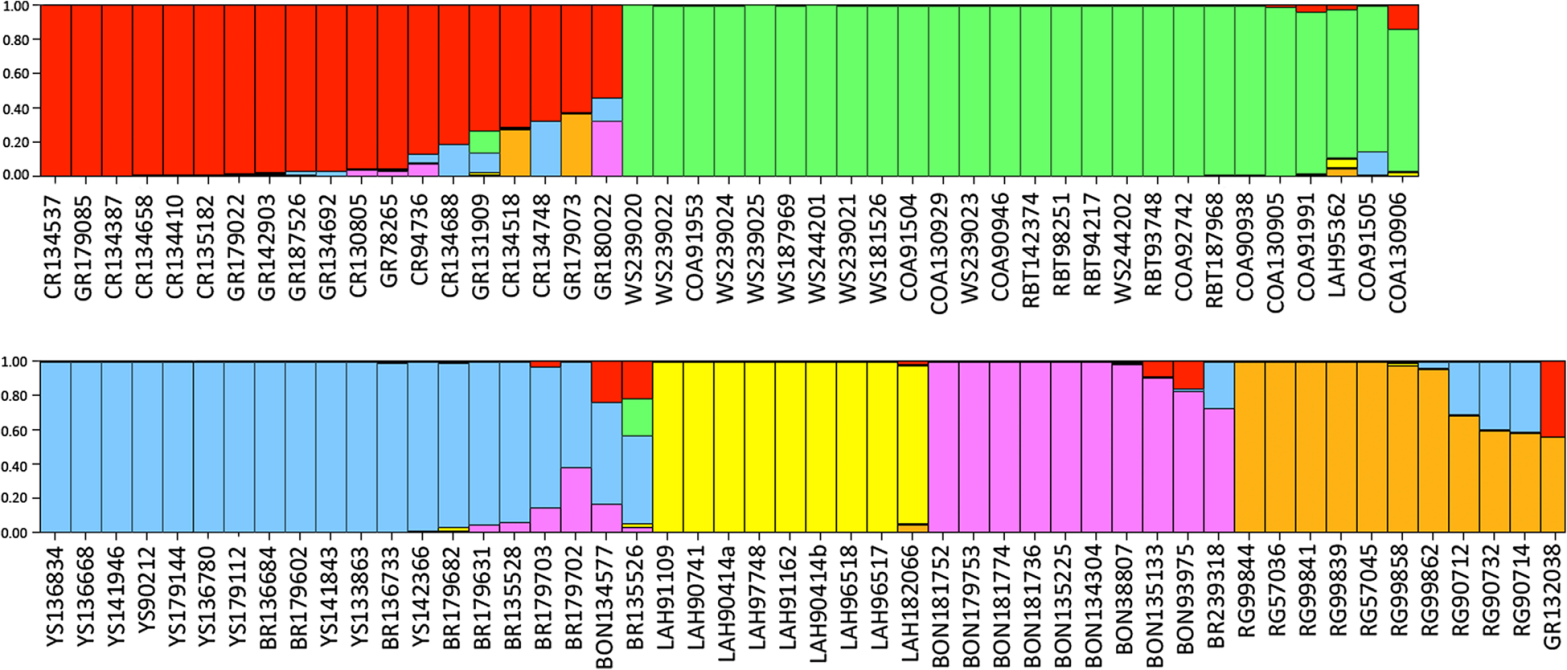
**Structure results.** Results of Structure analysis showing six unique populations of cutthroat trout and rainbow trout, Groups are shown as follows, beginning at the upper left and moving to the right: ColoradoRiver/greenback cutthroat (red), westslope/coastal/rainbow trout (green), Bear River/Yellowstone cutthroat (blue), Lahontan Basin cutthroat (yellow), Bonneville cutthroat (purple), and Rio Grande cutthroat (orange). Bars represent unique individuals, each of which is labeled using an abbreviation for the *a priori* subspecies designation (given in Figure 3) followed by that individual’s BYU identification number.

**Figure 6:**
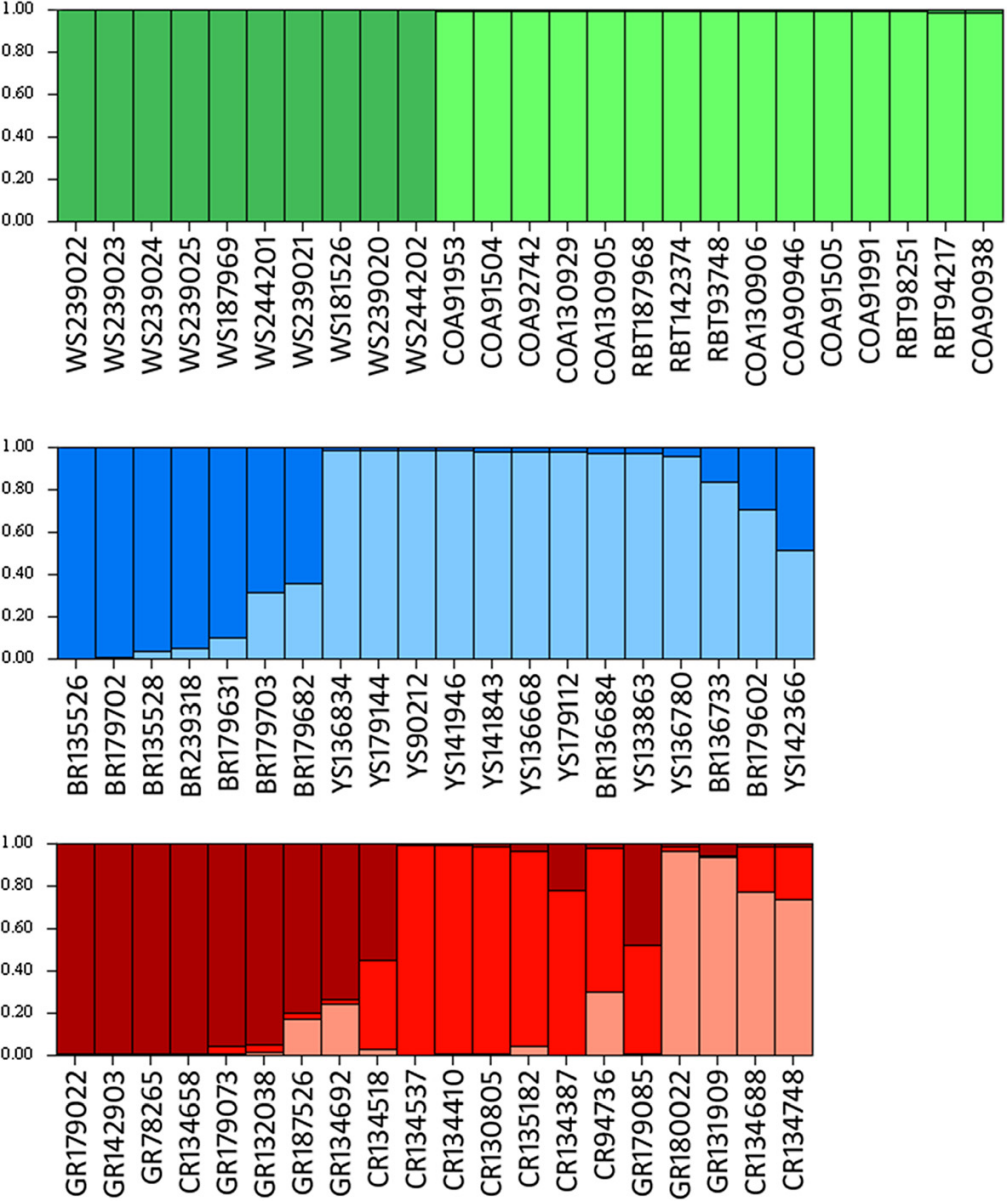
**Additional Structure results.** Results of secondary Structure analyses showing additional substructure in three groups that clustered as unique populations in the first set of analyses. Those groups are as follows: Westslope/Coastal/Rainbow, Bear River/Yellowstone, and Colorado River/Greenback. Secondary Structure analyses show two distinct populations in the westslope/coastal/rainbow group, two distinct populations in the Bear River/Yellowstone group, and three distinct populations in the Colorado River/greenback group.

Structure analysis and the results of Structure Harvester using the Evanno method revealed six distinct populations of cutthroat trout and rainbow trout, not the ten that we defined *a priori* (see Figure 5). Structure grouped Colorado River cutthroat trout and greenback cutthroat trout together rather than as distinct subspecies, and did the same for Bear River and Yellowstone cutthroat. Similarly, coastal cutthroat, westslope cutthroat and rainbow trout were also considered to be a single population in the Structure results. Several individuals, including those that did not cluster as expected on the phylogenetic network, showed evidence of hybridization, as illustrated by mixed colors in the bars representing those individuals (Figure 5). Further Structure analyses revealed two distinct populations within the westslope/coastal/rainbow group, with westslope cutthroat cleanly separating from coastal cutthroat and rainbow trout (Figure 6). Reanalysis of the Bear River/Yellowstone group resulted in two distinct populations of cutthroat trout, although the boundary between them was not as clean cut as the boundary between westslope cutthroat and the coastal cutthroat/rainbow trout group (Figure 6). Reanalysis of the Colorado River/Greenback group resulted in three distinct populations, although many individuals showed signs of genetic admixture, and all individuals that fell into the third group showed signs of hybridization with other subspecies of cutthroat trout (Figure 5 and Figure 6).

## Discussion

We have implemented genomic and bioinformatic protocols to discover over 28,000 putative SNPs among cutthroat trout subspecies. We were able to scrutinize these data to develop a SNP assay that contains 125 nuclear SNPs and is capable of differentiating most subspecies of cutthroat trout from one another and from rainbow trout. The SNP assay is a fast and cost-effective way to identify individuals of unknown genetic background to subspecies, and can be a valuable tool for management agencies in their efforts to evaluate the genetic structure of cutthroat trout populations in western North America, especially prior to constructing and implementing conservation plans.

These 125 putatively unlinked nuclear SNPs also allow for the detection of hybrid individuals, as evidenced by the results of our SNP diversity analyses. Indeed, the majority of the individuals that did not cluster as predicted in the phylogenetic network (see Results and Figure 3) carried a signature of genetic admixture between cutthroat trout subspecies in the results of Structure analysis (see Figure 5). For example, the three Rio Grande cutthroat individuals that clustered with Bear River and Yellowstone cutthroat in the phylogenetic network (RG90712, RG90714, and RG90732) carried Rio Grande cutthroat mtDNA haplotypes, but appear to carry both Rio Grande and Bear River-Yellowstone cutthroat alleles in the Structure bar graph (Figure 5). Moreover, the Lahontan Basin cutthroat trout individual that clustered with rainbow trout on the network did, in fact, carry rainbow trout mtDNA, and has mostly rainbow trout SNP alleles (Figure 5), thus illustrating a case where a probable misidentified specimen was successfully identified using the SNP markers (the sample was provided as a fin clip, so we were unable to go back to the voucher specimen to reassess the species identification). The Structure analysis was also useful in detecting other heterozygous/introgressed individuals, even if they did cluster with the pre-assigned subspecies on the network, as evidenced by a number of mixed bars representing Bear River, Bonneville, coastal, Colorado River, greenback, Lahontan Basin and Rio Grande cutthroat trout individuals (Figure 5).

The groups on the phylogenetic network (Figure 3) and in the PCoA results (Figure 4) accurately reflect what is known about phylogenetic relationships among cutthroat trout subspecies. It is generally accepted that coastal cutthroat trout was the first to branch off from the other cutthroat trout after the initial divergence between cutthroat and rainbow trout [25], so the apparent close genetic distance between coastal cutthroat trout and rainbow trout is not surprising. While there are many SNPs that differentiate cutthroat trout from rainbow trout [28,40,41], the majority of the SNP primers examined herein were chosen specifically to detect differences among subspecies of cutthroat trout. Of the 288 SNP loci for which primers were developed in this study, only six were selected that should have detected differences between rainbow and cutthroat trout, and not all of those amplified reliably (Additional file 2), so the inability to clearly differentiate the two species likely results from an ascertainment bias that is a direct result of that under-sampling.

It is somewhat surprising that the initial Structure analysis was unable to separate westslope cutthroat from coastal cutthroat and rainbow trout because both the phylogenetic network (Figure 3) and the PCoA results (Figure 4) show what appears to be a large genetic distance between westslope cutthroat and the other lineages. The fact that westslope cutthroat did separate from coastal cutthroat and rainbow trout in the second Structure analysis suggests that this might have been caused by the signal from a small number of alleles that were unique to westslope cutthroat trout (Additional file 2) being overridden by the signal from the larger data-set. Lahontan Basin cutthroat trout also exhibit large genetic distances between them and other cutthroat trout subspecies on our network (Figure 3), and they separated from the other subspecies in PCoA (Figure 4) and Structure analyses (Figure 5), which is consistent with previously published genetic distances and their hypothesized positions in published phylogenies [20,21,25,32]. The relatively smaller genetic distances between the other five lineages of cutthroat trout is also consistent with previously published data, and seems to correspond well with the evolution of these lineages in separate drainage basins. However, results of our Structure analyses show that these SNPs were initially unable to differentiate Bear River cutthroat from Yellowstone cutthroat, and only did so when the Bear River/Yellowstone subset was reanalyzed. The inability of even the second Structure analyses to cleanly separate these two subspecies may be because the final separation between Bear River and Yellowstone cutthroat likely corresponds with late Pleistocene events that resulted in the capture of the Bear River into the Bonneville Basin [26]. Gene flow was likely possible between Bear River and Yellowstone cutthroat trout populations up until the Bear River was diverted into the Bonneville Basin in the late Pleistocene [33-35]. It is possible that these two lineages have not been separated long enough for mutations to become fixed in each subspecies. It is also possible that our results are confounded by widespread stocking of Yellowstone cutthroat trout by management agencies prior to the recognition of unique lineages. Unfortunately, we are unable to distinguish between these two scenarios. Similarly, we were not able to cleanly differentiate Colorado River and greenback cutthroat trout from each other using this suite of SNPs, even after reanalyzing the reduced datasets using Structure. It is unclear whether the inability of these SNPs to differentiate between Colorado River and greenback cutthroat is because these subspecies have diverged too recently so alleles have not had time to reach fixation, or if there have been introductions resulting in introgressive hybridization in what we initially treated as non-admixed populations. Considering the close proximity of the drainages in which these subspecies reside and the frequency at which cutthroat trout were stocked in the past, the latter scenario is certainly plausible. A number of bars on the Structure bar graph represent Colorado River and greenback cutthroat trout individuals that appear to be hybrids (Figure 5), which lends support to the latter scenario. Clearly additional research is needed to resolve this issue.

Additional studies focused on SNP development in the Interior group of cutthroat trout (i.e., Bear River, Bonneville, Colorado River, Greenback, Rio Grande, and Yellowstone) are warranted, particularly when it comes to searching for fixed alleles between Bear River and Yellowstone cutthroat, and between Colorado River and greenback cutthroat trout, if they exist.

## Conclusions

The SNP markers reported here have added to a rapidly growing body of markers that can be used in cutthroat trout population genetic studies, and should be a valuable resource in future attempts to evaluate the genetic composition of cutthroat trout populations in western North America, including the detection of hybrids. These results reiterate that cutthroat trout subspecies are geographically and evolutionarily distinct, and ought to continue to be managed as such by state and federal agencies.

The method used to discover these SNP loci in cutthroat trout was developed for SNP discovery in the Eudicot genus *Amaranthus*[6]. Because the method was also successful for SNP discovery in something as evolutionary distant as cutthroat trout, it should be applicable to SNP discovery in many different kinds of non-model organisms.

## Competing interests

The authors declare that they have no competing interests.

## Authors’ contributions

DDH, DKS, JSKK, PJM and RPE designed the study. DDH, PJM, SMS and RBS generated the data. DBE, DDH, PJM and SMS analyzed the data. DDH, DKS, PJM, JSKK and RPE contributed to the writing of the manuscript. All authors have read and approved the final manuscript.

## Supplementary Material

Additional file 1 SNP primer table, SNP marker names, GenBank accession numbers, the type of polymorphism for each SNP, allele specific primers, common reverse primers and specific target amplification primers are listed herein. Click here for file

Additional file 2 **Characterization of each SNP locus, Major and minor alleles for all 125 SNP loci, along with the proportions of individuals within each *a priori *designated subspecies that carry the minor allele, as well as minor allele frequencies for each SNP locus are listed herein.** Click here for file

## References

[B1] Brumfeld RT Beerli P Nickerson DA Edwards SV The utility of single nucleotide polymorphisms in inferences of population history Trends Ecol Evol 2003 18 249 256 10.1016/S0169-5347(03)00018-1

[B2] Garvin MR Saitoh K Gharrett AJ Application of single nucleotide polymorphisms to non-model species: a technical review Mol Ecol Resour 2010 10 915 934 10.1111/j.1755-0998.2010.02891.x 21565101

[B3] Metzker ML Sequencing technologies - the next generation Nat Rev Genet 2010 11 31 46 10.1038/nrg2626 19997069

[B4] Helyar SJ Hemmer-Hansen J Bekkevold D Taylor MI Ogden R Limborg MT Cariani A Maes GE Diopere E Carvalho GR Nielsen EE Application of SNPs for population genetics of nonmodel organisms: new opportunities and challenges Mol Ecol Resour 2011 11 123 136 21429169 10.1111/j.1755-0998.2010.02943.x

[B5] Hale MC McCormick CR Jackson JR DeWoody JA Next-generation pyrosequencing of gonad transcriptomes in the polyploid lake sturgeon (Acipenser fulvescens): the relative merits of normalization and rarefaction in gene discovery BMC Genomics 2009 10 203 10.1186/1471-2164-10-203 19402907 PMC2688523

[B6] Maughan PJ Yourstone SM Jellen EN Udall JA Snp Discovery Via Genomic Reduction, Barcoding, And 454-pyrosequencing In Amaranth Plant Gen 2009 2 260 270 10.3835/plantgenome2009.08.0022

[B7] Wu X Ren C Joshi T Vuong T Xu D Nguyen HT SNP discovery by high-throughput sequencing in soybean BMC Genomics 2010 11 469 10.1186/1471-2164-11-469 20701770 PMC3091665

[B8] Oliver RE Lazo GR Lutz JD Rubenfield MJ Tinker NA Anderson JM Morehead NHW Adhikary D Jellen EN Maughan PJ Model SNP development for complex genomes based on hexaploid oat using high-throughput 454 sequencing technology BMC Genomics 2011 12 77 10.1186/1471-2164-12-77 21272354 PMC3041746

[B9] Williams LM Ma X Boyko AR Bustamante CD Oleksiak MF SNP identification, verification, and utility for population genetics in a non-model genus BMC Genet 2011 11 32 20433726 10.1186/1471-2156-11-32 PMC2874759

[B10] Aguilar A Garza JC Isolation of 15 single nucleotide polymorphisms from coastal steelhead, Oncorhynchus mykiss (Salmonidae) Mol Ecol Resour 2008 8 659 662 10.1111/j.1471-8286.2007.02038.x 21585863

[B11] Narum SR Banks M Beacham TD Bellinger MR Campbell MR Dekoning J Elz A Guthrie CM Kozfkay C Miller KM Differentiating salmon populations at broad and fine geographical scales with microsatellites and single nucleotide polymorphisms Mol Ecol 2008 17 3464 3477 19160476 10.1111/j.1365-294x.2008.03851.x

[B12] Sušnik S Sivka U Snoj A A set of nuclear DNA markers diagnostic for marble trout, Salmo marmoratus Aquaculture 2008 285 260 263 10.1016/j.aquaculture.2008.08.009

[B13] Castaño-Sánchez C Smith TP Wiedmann RT Vallejo RL Salem M Yao J Rexroad CE III Single nucleotide polymorphism discovery in rainbow trout by deep sequencing of a reduced representation library BMC Genomics C7 - 559 2009 10 559 10.1186/1471-2164-10-559 PMC2790473 19939274

[B14] Renaut S Nolte AW Rogers SM Derome N Bernatchez L SNP signatures of selection on standing genetic variation and their association with adaptive phenotypes along gradients of ecological speciation in lake whitefish species pairs (Coregonus spp.) Mol Ecol 2011 20 545 559 10.1111/j.1365-294X.2010.04952.x 21143332

[B15] Campbell NR Narum SR Development of 54 novel single-nucleotide polymorphism (SNP) assays for sockeye and coho salmon and assessment of available SNPs to differentiate stocks within the Columbia River Mol Ecol Resour 2011 11 20 30 21429160 10.1111/j.1755-0998.2011.02977.x

[B16] Fridjonsson O Olafsson K Tompsett S Bjornsdottir S Consuegra S Knox D de Leaniz CG Magnusdottir S Olafsdottir G Verspoor E Hjorleifsdottir S Detection and mapping of mtDNA SNPs in Atlantic salmon using high throughput DNA sequencing BMC Genomics 2011 12 179 10.1186/1471-2164-12-179 21473771 PMC3079667

[B17] Renaut S Nolte AW Bernatchez L Mining transcriptome sequences towards identifying adaptive single nucleotide polymorphisms in lake whitefish species pairs (Coregonus spp. Salmonidae) Mol Ecol 2010 19 115 131 20331775 10.1111/j.1365-294X.2009.04477.x

[B18] Seeb JE Pascal CE Grau ED Seeb LW Templin WD Harkins T Roberts SB Transcriptome sequencing and high-resolution melt analysis advance single nucleotide polymorphism discovery in duplicated salmonids Mol Ecol Resour 2011 11 335 348 10.1111/j.1755-0998.2010.02936.x 21429141

[B19] Lamaze FC Sauvage C Marie A Garant D Bernatchez L Dynamics of introgressive hybridization assessed by SNP population genomics of coding genes in stocked brook charr (Salvelinus fontinalis) Mol Ecol 2012 21 2877 2895 10.1111/j.1365-294X.2012.05579.x 22548328

[B20] Wilson WD Turner TF Phylogenetic analysis of the Pacific cutthroat trout (Oncorhynchus clarki ssp.: Salmonidae) based on partial mtDNA ND4 sequences: A closer look at the highly fragmented inland species Mol Phylogenet Evol 2009 52 406 415 10.1016/j.ympev.2009.03.018 19341807

[B21] Loxterman J Keeley E Watershed boundaries and geographic isolation: patterns of diversification in cutthroat trout from western North America BMC Evol Biol 2012 12 38 10.1186/1471-2148-12-38 22429757 PMC3320548

[B22] Trotter PC Behnke RJ The case for Humboldtensis: A subspecies name for the indigenous cutthroat trout (Oncorhynchus clarkii) of the Humboldt River, Upper Quinn River, and Coyote Basin drainages, Nevada and Oregon Western North American Naturalist 2008 68 58 65 10.3398/1527-0904(2008)68[58:TCFHAS]2.0.CO;2

[B23] Montgomery MR Many rivers to cross: of good running water, native trout, and the remains of wilderness 1995 New York: Touchstone

[B24] Behnke RJ Native trout of western North America American Fisheries Society Monograph 1992 6 1 275

[B25] Behnke RJ Trout and Salmon of North America 2002 New York, NY: The Free Press

[B26] Johnson AE Resolving phylogenetic relationships of selected cutthroat trout subspecies, Oncorhynchus clarki (Salmonidae) 2005 Department of Biology: Brigham Young University

[B27] Busack CA Gall GAE Introgressive hybridization in populations of Paiute cutthroat trout (Salmo-clarki-seleniris) Can J Fish Aquat Sci 1981 38 939 951 10.1139/f81-127

[B28] Finger AJ Stephens MR Clipperton NW May B Six diagnostic single nucleotide polymorphism markers for detecting introgression between cutthroat and rainbow trouts Mol Ecol Resour 2009 9 759 763 10.1111/j.1755-0998.2009.02532.x 21564737

[B29] Behnke RJ Soltz N Systematic and zoogeographical interpretation of Great Basin trouts Fishes In North American Deserts 1981 New York: John Wiley & Sons 95 124

[B30] Loudenslager EJ Gall GAE Geographic Patterns of Protein Variation and Subspeciation in Cutthroat Trout, Salmo clarki Syst Zool 1980 29 27 42 10.2307/2412624

[B31] Martin MA Shiozawa DK Loudenslager EJ Jensen JN Electrophoretic study of cutthroat trout populations in Utah Great Basin Naturalist 1985 45 677 687

[B32] Smith GR Dowling T Gobalet K Lugaski T Shiozawa DK Evans RP Biogeography and timing of evolutionary events among Great Basin fishes Great Basin Aquatic Systems History 2002 33 Washington, D. C: Smithsonian Institution Press 175 234 Smithsonian Contributions to the Earth Sciences

[B33] Malde HE The catastrophic late Pleistocene Bonneville Flood in the Snake River Plain, Idaho 1968 Washington, D. C: United States Government Printing Office

[B34] Bouchard DP Kaufman DS Hochberg A Quade J Quaternary history of the Thatcher Basin, Idaho, reconstructed from the Sr-87/Sr-86 and amino acid composition of lacustrine fossils: implications for the diversion of the Bear River into the Bonneville Basin Palaeogeogr Palaeoclimatol Palaeoecol 1998 141 95 114 10.1016/S0031-0182(98)00005-4

[B35] Johnson JB Evolution after the flood: phylogeography of the desert fish Utah Chub (Gila atraria) Evolution 2002 56 948 960 12093030 10.1111/j.0014-3820.2002.tb01407.x

[B36] Metcalf JL Love Stowell S Kennedy CM Rogers KB McDonald D Epp J Keepers K Cooper A Austin JJ Martin AP Historical stocking data and 19th century DNA reveal human-induced changes to native diversity and distribution of cutthroat trout Mol Ecol 2012 21 5194 5207 10.1111/mec.12028 22998121

[B37] Allendorf FW Leary RF Conservation and distribution of genetic variation in a polytypic species the cutthroat trout Conserv Biol 1988 2 170 184 10.1111/j.1523-1739.1988.tb00168.x

[B38] Trotter PC Cutthroat native trout of the west 2008 2 Berkeley, CA: University of California Press

[B39] Hohenlohe PA Amish SJ Catchen JM Allendorf FW Luikart G Next-generation RAD sequencing identifies thousands of SNPs for assessing hybridization between rainbow and westslope cutthroat trout Mol Ecol Resour 2011 11 117 122 21429168 10.1111/j.1755-0998.2010.02967.x

[B40] McGlauflin MT Smith MJ Wang JT Young SF Chen N Lee YC Pascal C Seeb LW Stevens J Seeb JE High-Resolution Melting Analysis for the Discovery of Novel Single-Nucleotide Polymorphisms in Rainbow and Cutthroat Trout for Species Identification Trans Am Fish Soc 2011 139 676 684

[B41] Pritchard VL Abadía-Cardoso A Garza JC Discovery and characterization of a large number of diagnostic markers to discriminate Oncorhynchus mykiss and O. clarkii Mol Ecol Resour 2012 12 918 931 10.1111/j.1755-0998.2012.03149.x 22591214

[B42] Amish SJ Hohenlohe PA Painter S Leary RF Muhlfeld C Allendorf FW Luikart G RAD sequencing yields a high success rate for westslope cutthroat and rainbow trout species-diagnostic SNP assays Mol Ecol Resour 2012 12 653 660 10.1111/j.1755-0998.2012.03157.x 22672623

[B43] Kalinowski ST Novak BJ Drinan DP Jennings R Vu NV Diagnostic single nucleotide polymorphisms for identifying westslope cutthroat trout (Oncorhynchus clarki lewisi), Yellowstone cutthroat trout (Oncorhynchus clarkii bouvieri) and rainbow trout (Oncorhynchus mykiss) Mol Ecol Resour 2011 11 389 393 10.1111/j.1755-0998.2010.02932.x 21429151

[B44] Harwood AS Phillips RB A suite of twelve single nucleotide polymorphism markers for detecting introgression between cutthroat and rainbow trout Mol Ecol Resour 2011 11 382 385 10.1111/j.1755-0998.2010.02930.x 21429149

[B45] Campbell NR Amish SJ Pritchard VL McKelvey KS Young MK Schwartz MK Garza JC Luikart G Narum SR Development and evaluation of 200 novel SNP assays for population genetic studies of westslope cutthroat trout and genetic identification of related taxa Mol Ecol Resour 2012 12 942 949 10.1111/j.1755-0998.2012.03161.x 22697369

[B46] Smit AFA Hubley R Green P RepeatMasker Open-3.0 1996–2010 2010 http://www.repeatmasker.org

[B47] KBiosciences PrimerPicker Lite for KASPar v.0.26 2009 Hoddesdon, UK: KBiosciences Ltd

[B48] Wang J Lin M Crenshaw A Hutchinson A Hicks B Yeager M Berndt S Huang W-Y Hayes RB Chanock SJ High-throughput single nucleotide polymorphism genotyping using nanofluidic Dynamic Arrays BMC Genomics 2009 10 10.1186/1471-2164-10-561 PMC2789104 19943955

[B49] Maughan PJ Smith SM Fairbanks DJ Jellen EN Development, Characterization, and Linkage Mapping of Single Nucleotide Polymorphisms in the Grain Amaranths (Amaranthus sp.) Plant Gen 2011 4 92 101 10.3835/plantgenome2010.12.0027

[B50] Fluidigm Fluidigm SNP Genotyping Analysis v.3.0.2 2011 South San Francisco, CA: Fluidigm Corporation

[B51] Huson DH Bryant D Application of Phylogenetic Networks in Evolutionary Studies Mol Biol Evol 2006 23 254 267 16221896 10.1093/molbev/msj030

[B52] Goslee SC Urban DL The ecodist package for dissimilarity-based analysis of ecological data J Stat Softw 2007 22 1 19

[B53] R Development Core Team. R A language and environment for statistical computing 2012 Vienna, Austria: R Foundation for Statistical Computing http://www.R-project.org

[B54] Pritchard JK Stephens M Donnelly P Inference of Population Structure Using Multilocus Genotype Data Genetics 2000 155 945 959 10835412 10.1093/genetics/155.2.945 PMC1461096

[B55] Earl DA VonHoldt BM STRUCTURE HARVESTER: a website and program for visualizing STRUCTURE output and implementing the Evanno method Conserv Genet Resour 2012 4 359 361 10.1007/s12686-011-9548-7

