# Supplementary material for: Single nucleotide polymorphism discovery in cutthroat trout subspecies using genome reduction, barcoding, and 454 pyro-sequencing
Source: BMC Genomics. 2012 Dec 23;13:724. doi: 10.1186/1471-2164-13-724 (PMC3549761; doi:10.1186/1471-2164-13-724)
Supplement: Additional file 1 — SNP primer table, SNP marker names, GenBank accession numbers, the type of polymorphism for each SNP, allele specific primers, common reverse primers and specific target amplification primers are listed herein. [file 1471-2164-13-724-S1.docx]

**Additional File 1**: SNP Primer Table. SNP marker name (numbered by contig and base position), GenBank accession number (NCBI_ss#), SNP polymorphism type, KASPar^TM^ primer sequences (allele specific primer 1 [A1], allele specific primer 2 [A2], common reverse primer, and specific target amplification [STA] primer) for all 125 functional SNP assays. The first 21 bases of primers A1 and A2 are the sequence tags used in the KASPar^TM^ secondary PCR reaction and are shown in bold font.

| SNP ID | NCBI_ss# | SNP Type | A1 Primer (5’ – 3’) | A2 Primer (5’ – 3’) | Common Reverse Primer (5’-3’) | STA Primer (5’ – 3’) |
| --- | --- | --- | --- | --- | --- | --- |
| Oc18318_297 | 538828736 | G/T | **GAAGGTGACCAAGTTCATGCT**GATCATCAATGGGAAAGAAATTCGGC | **GAAGGTCGGAGTCAACGGATT**GATCATCAATGGGAAAGAAATTCGGA | CGAGTTAGACTCTGGCATTCACTCAA | GATCATCAATGGGAAAGAAATTCGG |
| Oc02855_246 | 538828738 | C/T | **GAAGGTGACCAAGTTCATGCT**CTCCACAGAACCGTCTCG | **GAAGGTCGGAGTCAACGGATT**GCTCTCCACAGAACCGTCTCA | TGCAGCGGTCACTCGGCTGTTT | CTCCACAGAACCGTCTC |
| Oc00719_477 | 538828740 | G/T | **GAAGGTGACCAAGTTCATGCT**GTATCGCTAAATACTGCAGCAATTTTTC | **GAAGGTCGGAGTCAACGGATT**ATGTATCGCTAAATACTGCAGCAATTTTTA | ATATCTGTGGATTCCTGACAGTACGTA | GTATCGCTAAATACTGCAGCAATTTTT |
| Oc01000_503 | 538828742 | A/C | **GAAGGTGACCAAGTTCATGCT**GGTTATTGTATATTCAAACTAATAACGCAAATT | **GAAGGTCGGAGTCAACGGATT**GTTATTGTATATTCAAACTAATAACGCAAATG | TGCTGGAGGCAGGTAGATTGTTGTA | GGTTATTGTATATTCAAACTAATAACGCAAAT |
| Oc04520_300 | 538828743 | A/G | **GAAGGTGACCAAGTTCATGCT**TGAACTGGTTGGAACTGAAAGACAAT | **GAAGGTCGGAGTCAACGGATT**GAACTGGTTGGAACTGAAAGACAAC | GCACAGTGGTCTTTTTCATAATAGAAGAAT | TGAACTGGTTGGAACTGAAAGACAA |
| Oc07619_342 | 538828745 | A/T | **GAAGGTGACCAAGTTCATGCT**GCAATGAAGAAGTACGGCCATCT | **GAAGGTCGGAGTCAACGGATT**GCAATGAAGAAGTACGGCCATCA | CACTTCAACTATGACAGACAAAATGAGAAA | GCAATGAAGAAGTACGGCCATC |
| Oc02334_453 | 538828747 | A/G | **GAAGGTGACCAAGTTCATGCT**GAATGTTGAATTGATAGAGCTCTGCTTT | **GAAGGTCGGAGTCAACGGATT**AATGTTGAATTGATAGAGCTCTGCTTC | GGAATGAGTTAGTTACCACACAATGCAAT | GAATGTTGAATTGATAGAGCTCTGCTT |
| Oc07137_365 | 538828749 | C/T | **GAAGGTGACCAAGTTCATGCT**GAGTGGTACTCAGTCACGTGTTG | **GAAGGTCGGAGTCAACGGATT**AGAGTGGTACTCAGTCACGTGTTA | GGCTATACCATGTTAGTGGTAAATTGAATA | GAGTGGTACTCAGTCACGTGTT |
| Oc11603_128 | 538828750 | A/C | **GAAGGTGACCAAGTTCATGCT**AACCTCTTCATTCAATATTCAACATTCAGT | **GAAGGTCGGAGTCAACGGATT**CCTCTTCATTCAATATTCAACATTCAGG | CCCAATGCACGCTTTTGGCTTCAT | AACCTCTTCATTCAATATTCAACATTCAG |
| Oc01121_395 | 538828752 | G/A | **GAAGGTGACCAAGTTCATGCT**GTCGCATCTATTTGTGATGCAAGTC | **GAAGGTCGGAGTCAACGGATT**AAGTCGCATCTATTTGTGATGCAAGTT | GTATATGCTTCTAAAAGCCAATGAGGAGAT | GTCGCATCTATTTGTGATGCAAGT |
| Oc01727_205 | 538828754 | C/T | **GAAGGTGACCAAGTTCATGCT**CCAGAATTCGGACACGGCCAG | **GAAGGTCGGAGTCAACGGATT**CCAGAATTCGGACACGGCCAA | GTTAGTAAAGCGCTAACTGACCGTGAT | CCAGAATTCGGACACGGCCA |
| Oc04584_342 | 538828756 | G/T | **GAAGGTGACCAAGTTCATGCT**CTTATTCAATGGATTTTGATGACACATC | **GAAGGTCGGAGTCAACGGATT**CTCTTATTCAATGGATTTTGATGACACATA | CACTGTGCAGGAATGAACGTCTGTT | CTTATTCAATGGATTTTGATGACACAT |
| Oc00002_837 | 538828758 | T/C | **GAAGGTGACCAAGTTCATGCT**CATACCATTTGGCCGGAACTGAATA | **GAAGGTCGGAGTCAACGGATT**ATACCATTTGGCCGGAACTGAATG | CACGTTAAAAATGTACCGGGCCGAT | CATACCATTTGGCCGGAACTGAAT |
| Oc07779_247 | 538828759 | C/A | **GAAGGTGACCAAGTTCATGCT**CGTAAACCTTATAAGTAGCACTTAATAAAATG | **GAAGGTCGGAGTCAACGGATT**ACGTAAACCTTATAAGTAGCACTTAATAAAATT | GCACTTTGTGACACTCTCCTGATGTA | CGTAAACCTTATAAGTAGCACTTAATAAAAT |
| Oc22333_295 | 538828761 | A/T | **GAAGGTGACCAAGTTCATGCT**AAGTAAACATGATCAGTTAACAGTACATCT | **GAAGGTCGGAGTCAACGGATT**AAGTAAACATGATCAGTTAACAGTACATCA | ATCCTTTTTGGGCTCTGCCTGTCAA | AAGTAAACATGATCAGTTAACAGTACATC |
| Oc06689_176 | 538828763 | T/A | **GAAGGTGACCAAGTTCATGCT**GCAATCCAGCGTATGGGTAAGATAA | **GAAGGTCGGAGTCAACGGATT**GCAATCCAGCGTATGGGTAAGATAT | GTAAAGATCTATGCAGTTGATACTGGAGAA | GCAATCCAGCGTATGGGTAAGATA |
| Oc04368_474 | 538828765 | C/G | **GAAGGTGACCAAGTTCATGCT**GGGTGAAATGTGTGGGCTCAG | **GAAGGTCGGAGTCAACGGATT**GGGTGAAATGTGTGGGCTCAC | CAAGCATTTGGCTTCCCTTGATACAAATA | GGGTGAAATGTGTGGGCTCA |
| Oc00162_353 | 538828766 | G/T | **GAAGGTGACCAAGTTCATGCT**GGGAGGACCTTAGGAACATTCC | **GAAGGTCGGAGTCAACGGATT**CGGGAGGACCTTAGGAACATTCA | AGTGAGAGAACCGTTAGGGTGAGAA | GGGAGGACCTTAGGAACATTC |
| Oc06563_94 | 538828768 | G/A | **GAAGGTGACCAAGTTCATGCT**CTACATTCAACACATGACCACTCC | **GAAGGTCGGAGTCAACGGATT**CTCTACATTCAACACATGACCACTCT | GGAGTGGTCATGTTGAATGTAGAGACA | CTACATTCAACACATGACCACTC |
| Oc01301_225 | 538828769 | T/G | **GAAGGTGACCAAGTTCATGCT**GCCACCGGAAGAGCACGGA | **GAAGGTCGGAGTCAACGGATT**CCACCGGAAGAGCACGGC | CGTCATGCCGTTGTCATGTTACGTT | GCCACCGGAAGAGCACGG |
| Oc01849_333 | 538828771 | C/T | **GAAGGTGACCAAGTTCATGCT**CGTTCGTAGCTCCAGACTCATG | **GAAGGTCGGAGTCAACGGATT**ACGTTCGTAGCTCCAGACTCATA | GCAGACAACCCTTCAAGCCTACATT | CGTTCGTAGCTCCAGACTCAT |
| Oc04318_267 | 538828773 | A/T | **GAAGGTGACCAAGTTCATGCT**CAAAGATGAGGAGAATTATCATATGTGATT | **GAAGGTCGGAGTCAACGGATT**CAAAGATGAGGAGAATTATCATATGTGATA | GGCACAGGGAGACGACTTTAATCAT | CAAAGATGAGGAGAATTATCATATGTGAT |
| Oc04332_250 | 538828775 | T/C | **GAAGGTGACCAAGTTCATGCT**CATAGGCGTAACACAGCACCGA | **GAAGGTCGGAGTCAACGGATT**ATAGGCGTAACACAGCACCGG | CATAACCATTCTCAGGTCAGAAGAATGTT | CATAGGCGTAACACAGCACCG |
| Oc13395_65 | 538828776 | C/T | **GAAGGTGACCAAGTTCATGCT**AGATAAAGAAGACAACTGGCAGCAG | **GAAGGTCGGAGTCAACGGATT**GAGATAAAGAAGACAACTGGCAGCAA | TTTGTGACATGTGGAATAAAAGCCACTATT | AGATAAAGAAGACAACTGGCAGCA |
| Oc02233_468 | 538828778 | T/A | **GAAGGTGACCAAGTTCATGCT**ATTTTTATACTTTCAGGTGATGATTTAAATGTA | **GAAGGTCGGAGTCAACGGATT**TTTATACTTTCAGGTGATGATTTAAATGTT | GTGTGATGTTCGAATAACATTTACAAACAA | ATTTTTATACTTTCAGGTGATGATTTAAATGT |
| Oc04162_306 | 538828780 | C/T | **GAAGGTGACCAAGTTCATGCT**TGCGTCAAACGTTAGGTTTG | **GAAGGTCGGAGTCAACGGATT**AATGCTTGCGTCAAACGTTAGGTTTA | AAATGCAGAGAAGAGTAGTTCCAGCATTA | TGCGTCAAACGTTAGGTTT |
| Oc03207_330 | 538828781 | T/A | **GAAGGTGACCAAGTTCATGCT**GTGGCACAAATCCCACAGATCGA | **GAAGGTCGGAGTCAACGGATT**GTGGCACAAATCCCACAGATCGT | CATTTCTTGTGAAGCTTAACAAAGTCAGTT | GTGGCACAAATCCCACAGATCG |
| Oc02284_320 | 538828783 | G/A | **GAAGGTGACCAAGTTCATGCT**GTTCATTGTCTTAGCTTATTCAAGTAGC | **GAAGGTCGGAGTCAACGGATT**GTTCATTGTCTTAGCTTATTCAAGTAGT | GATTCCAAAATCTGACTTCCTGAGTTGAA | GTTCATTGTCTTAGCTTATTCAAGTAG |
| Oc02956_408 | 538828785 | T/C | **GAAGGTGACCAAGTTCATGCT**ATCTACTATTCGGAAGTTACCCCATTA | **GAAGGTCGGAGTCAACGGATT**CTACTATTCGGAAGTTACCCCATTG | CCCTATTTATCTAACCTTCCTTAACCCTT | ATCTACTATTCGGAAGTTACCCCATT |
| Oc09421_125 | 538828787 | T/A | **GAAGGTGACCAAGTTCATGCT**TAGAAAGTATATCCACTACATGAACAAA | **GAAGGTCGGAGTCAACGGATT**CTTAGAAAGTATATCCACTACATGAACAAT | TCCTTTTTCATATTTCATGCTCACCGCAT | TAGAAAGTATATCCACTACATGAACAA |
| Oc04740_310 | 538828788 | T/C | **GAAGGTGACCAAGTTCATGCT**TGAATTGTTTTCTCATCATGTTGAGACAA | **GAAGGTCGGAGTCAACGGATT**GAATTGTTTTCTCATCATGTTGAGACAG | AGGAAGCTACAGGCGCATATGTCTT | TGAATTGTTTTCTCATCATGTTGAGACA |
| Oc09353_200 | 538828790 | T/C | **GAAGGTGACCAAGTTCATGCT**ACGGAGAGCAATGTACAATATGGCA | **GAAGGTCGGAGTCAACGGATT**GGAGAGCAATGTACAATATGGCG | GGTGTCTCCCATACACTTACCACAA | ACGGAGAGCAATGTACAATATGGC |
| Oc00078_436 | 538828792 | A/C | **GAAGGTGACCAAGTTCATGCT**GAAACACATTGGGACAACGTATGTATTT | **GAAGGTCGGAGTCAACGGATT**AAACACATTGGGACAACGTATGTATTG | CACCACTTGAATTCCCCCTACCATT | GAAACACATTGGGACAACGTATGTATT |
| Oc02899_311 | 538854200 | T/C | **GAAGGTGACCAAGTTCATGCT**AATTGGTCCTTTGACCAATCAGATCAA | **GAAGGTCGGAGTCAACGGATT**GGTCCTTTGACCAATCAGATCAG | CACAGGCAGCCCAATTCTGATCTTT | AATTGGTCCTTTGACCAATCAGATCA |
| Oc01496_132 | 538828794 | G/A | **GAAGGTGACCAAGTTCATGCT**AGCAGCACTCTGTCACTGTTCC | **GAAGGTCGGAGTCAACGGATT**AGCAGCACTCTGTCACTGTTCT | AGCTTTTCAGCTCGGGGCTATGAT | AGCAGCACTCTGTCACTGTTC |
| Oc15008_273 | 538828795 | T/A | **GAAGGTGACCAAGTTCATGCT**GTACTGTATGTATTCTGCACATCCATTA | **GAAGGTCGGAGTCAACGGATT**GTACTGTATGTATTCTGCACATCCATTT | TGTTCCACTGTGATCAACTGCGCTA | GTACTGTATGTATTCTGCACATCCATT |
| Oc22659_144 | 538828797 | A/G | **GAAGGTGACCAAGTTCATGCT**ACCGTCTATGATCTACGGGCGT | **GAAGGTCGGAGTCAACGGATT**CCGTCTATGATCTACGGGCGC | GCAGCTGTAATAAGGGACATAAAACGTAA | ACCGTCTATGATCTACGGGCG |
| Oc27906_245 | 538828799 | A/T | **GAAGGTGACCAAGTTCATGCT**ACTTGAAAAATGATGTGACATATCTGTACT | **GAAGGTCGGAGTCAACGGATT**ACTTGAAAAATGATGTGACATATCTGTACA | GTTGGTTTGGTTGACTGTATGTTAGAGTT | ACTTGAAAAATGATGTGACATATCTGTAC |
| Oc23835_105 | 538828800 | A/C | **GAAGGTGACCAAGTTCATGCT**GCGTATGTGATAAATAGAATATTGAACAATTT | **GAAGGTCGGAGTCAACGGATT**GCGTATGTGATAAATAGAATATTGAACAATTG | ATTTAAAATGTCCATCATGAAACATTTCCT | GCGTATGTGATAAATAGAATATTGAACAATT |
| Oc12661_311 | 538828802 | A/G | **GAAGGTGACCAAGTTCATGCT**GATACGTATAACAACACAAAGAGCATGT | **GAAGGTCGGAGTCAACGGATT**ATACGTATAACAACACAAAGAGCATGC | TCATTATCAATGACGACCCTGGTTTGTA | GATACGTATAACAACACAAAGAGCATG |
| Oc25168_210 | 538828804 | C/A | **GAAGGTGACCAAGTTCATGCT**GAGAGAGAGATGCAGTTCACCTG | **GAAGGTCGGAGTCAACGGATT**GAGAGAGAGATGCAGTTCACCTT | AGTAATTTTGAACCGATGACCAAATCTCAT | GAGAGAGAGATGCAGTTCACCT |
| Oc15656_263 | 538828805 | C/A | **GAAGGTGACCAAGTTCATGCT**AGGGTCTCTTTTCCAGGCTTAAAAG | **GAAGGTCGGAGTCAACGGATT**AAGGGTCTCTTTTCCAGGCTTAAAAT | GGATTGACAGCATGGCCAATGGTAT | AGGGTCTCTTTTCCAGGCTTAAAA |
| Oc17530_158 | 538828807 | G/A | **GAAGGTGACCAAGTTCATGCT**GTTCCCTGCACAAGAATAAAGGAC | **GAAGGTCGGAGTCAACGGATT**CGTTCCCTGCACAAGAATAAAGGAT | GTTAAATGTCTGTTTGTGTTGGGCAGT | GTTCCCTGCACAAGAATAAAGGA |
| Oc12934_230 | 538828808 | T/A | **GAAGGTGACCAAGTTCATGCT**ATGCATAACACTTCTATCTTTTATCCATGA | **GAAGGTCGGAGTCAACGGATT**ATGCATAACACTTCTATCTTTTATCCATGT | GCAGAGCCACATCAATTTACAGAA | ATGCATAACACTTCTATCTTTTATCCATG |
| Oc11988_119 | 538828810 | A/G | **GAAGGTGACCAAGTTCATGCT**TAACAAAACTCCTCACCACAAGACAT | **GAAGGTCGGAGTCAACGGATT**AACAAAACTCCTCACCACAAGACAC | TGTTTGGTTAAGACGCGTCCATATGTTT | TAACAAAACTCCTCACCACAAGACA |
| Oc24645_268 | 538828812 | G/C | **GAAGGTGACCAAGTTCATGCT**CAACTTCTGAAGTTCGAAACAC | **GAAGGTCGGAGTCAACGGATT**GCTCAACTTCTGAAGTTCGAAACAG | CATGCAAAGTCATGGGGCGTGCAT | CAACTTCTGAAGTTCGAAACA |
| Oc25786_318 | 538828813 | T/A | **GAAGGTGACCAAGTTCATGCT**AATTTGTTTATTAGTCTATTAACTGACTCCATA | **GAAGGTCGGAGTCAACGGATT**AATTTGTTTATTAGTCTATTAACTGACTCCATT | CAGCAATAACTTATACTTGCTGGCACATT | AATTTGTTTATTAGTCTATTAACTGACTCCAT |
| Oc17643_137 | 538828815 | A/G | **GAAGGTGACCAAGTTCATGCT**ACTGTAAGCCATGTTACAATTCCCAT | **GAAGGTCGGAGTCAACGGATT**CTGTAAGCCATGTTACAATTCCCAC | TACAATGTGTCACGCACACTGGGTT | ACTGTAAGCCATGTTACAATTCCCA |
| Oc19939_384 | 538828816 | T/A | **GAAGGTGACCAAGTTCATGCT**GCAATCCAGCGTATGGGTAAGATAA | **GAAGGTCGGAGTCAACGGATT**GCAATCCAGCGTATGGGTAAGATAT | GTAAAGATCTATGCAGTTGATACTGGAGAA | GCAATCCAGCGTATGGGTAAGATA |
| Oc07334_176 | 538828818 | A/C | **GAAGGTGACCAAGTTCATGCT**CCTCCACCGAGACCAGATCATAT | **GAAGGTCGGAGTCAACGGATT**CTCCACCGAGACCAGATCATAG | ATTGCACTCACCAAGCGAAGTTTGATTAA | CCTCCACCGAGACCAGATCATA |
| Oc00345_62 | 538854243 | T/G | **GAAGGTGACCAAGTTCATGCT**CCCTTAAACAATTCCCACAGTAA | **GAAGGTCGGAGTCAACGGATT**CCCTTAAACAATTCCCACAGTAC | GGGACACTATGTAGTTCTTGAAATAATGTA | CCCTTAAACAATTCCCACAGTA |
| Oc32430_172 | 538828821 | C/T | **GAAGGTGACCAAGTTCATGCT**ATTTCTGAAGTGGTGTGAGAGAGAG | **GAAGGTCGGAGTCAACGGATT**ATTTCTGAAGTGGTGTGAGAGAGAA | CACATGACCATAATTAGGTAGCATCTGAA | ATTTCTGAAGTGGTGTGAGAGAGA |
| Oc35801_240 | 538828823 | C/T | **GAAGGTGACCAAGTTCATGCT**ACACAGATCTGGGGACCTGG | **GAAGGTCGGAGTCAACGGATT**CTACACAGATCTGGGGACCTGA | GGACCTTGACAGCTGCACGGAA | ACACAGATCTGGGGACCTG |
| Oc06387_477 | 538828825 | C/T | **GAAGGTGACCAAGTTCATGCT**CCAATTCCTCATCATGATTGATCCG | **GAAGGTCGGAGTCAACGGATT**ACCAATTCCTCATCATGATTGATCCA | GTTTTCAAATATCGAGATGATTCGAA | CCAATTCCTCATCATGATTGATCC |
| Oc15945_33 | 538828827 | A/G | **GAAGGTGACCAAGTTCATGCT**AATGGACCCAGCGACTTCGGAT | **GAAGGTCGGAGTCAACGGATT**GGACCCAGCGACTTCGGAC | GCTCCCTGGATCTCGACGGCT | AATGGACCCAGCGACTTCGGA |
| Oc06930_61 | 538828829 | G/T | **GAAGGTGACCAAGTTCATGCT**GAAGAAGAAGAGCATGAAATTCTATCTTC | **GAAGGTCGGAGTCAACGGATT**GAAGAAGAAGAGCATGAAATTCTATCTTA | TGAGTTTGGCTCAGGGGGCCAA | GAAGAAGAAGAGCATGAAATTCTATCTT |
| Oc20487_212 | 538828831 | C/T | **GAAGGTGACCAAGTTCATGCT**AGATCGTCATGTTATCCAG | **GAAGGTCGGAGTCAACGGATT**AGATCGTCATGTTATCCAA | TGATGCATGTGTAACAAAGGTAATTAGGC | AGATCGTCATGTTATCCA |
| Oc21247_311 | 538828832 | A/G | **GAAGGTGACCAAGTTCATGCT**AACATACTGGTTGCATGCTGTCAGT | **GAAGGTCGGAGTCAACGGATT**CATACTGGTTGCATGCTGTCAGC | CAGAGCAGAAACATGGTCAGGTTGAA | AACATACTGGTTGCATGCTGTCAG |
| Oc25620_232 | 538828834 | T/C | **GAAGGTGACCAAGTTCATGCT**TATGTTTGAGAAGAGCGATCA | **GAAGGTCGGAGTCAACGGATT**GCTTATGTTTGAGAAGAGCGATCG | GAATGTGTCAAACCTGTCATAGCGGAA | TATGTTTGAGAAGAGCGATC |
| Oc15641_178 | 538828835 | A/C | **GAAGGTGACCAAGTTCATGCT**TAGATCTCAGGCTCTCGTTCATCT | **GAAGGTCGGAGTCAACGGATT**GATCTCAGGCTCTCGTTCATCG | TCACAGATTCCCGAAGCCCTCATAT | TAGATCTCAGGCTCTCGTTCATC |
| Oc13704_250 | 538828837 | C/T | **GAAGGTGACCAAGTTCATGCT**GCGGAGAGAGTGCGTGGAG | **GAAGGTCGGAGTCAACGGATT**GGCGGAGAGAGTGCGTGGAA | AGCCACAAATGAATTTGAACAACCTGTC | GCGGAGAGAGTGCGTGGA |
| Oc38332_102 | 538828839 | T/G | **GAAGGTGACCAAGTTCATGCT**GAAAAGCTTTGATCTCACAATGAACATTTTA | **GAAGGTCGGAGTCAACGGATT**AAAAGCTTTGATCTCACAATGAACATTTTC | CTGTTCTGCCTACAGACCACCTATA | GAAAAGCTTTGATCTCACAATGAACATTTT |
| Oc21532_151 | 538828840 | A/C | **GAAGGTGACCAAGTTCATGCT**GCATCAGCTATCCAACACAAACCT | **GAAGGTCGGAGTCAACGGATT**CATCAGCTATCCAACACAAACCG | GCAGGATACAGAGGTAAAGCATTAAGATA | GCATCAGCTATCCAACACAAACC |
| Oc29764_214 | 538828842 | C/T | **GAAGGTGACCAAGTTCATGCT**AACCTAACATCTAATGACAAAACATACTG | **GAAGGTCGGAGTCAACGGATT**CAACCTAACATCTAATGACAAAACATACTA | AAAACCATCTGCCACTTATTCCTGCAAA | AACCTAACATCTAATGACAAAACATACT |
| Oc29764_66 | 538828843 | T/G | **GAAGGTGACCAAGTTCATGCT**AATTAGACTTACCCCACAGCAAGAATA | **GAAGGTCGGAGTCAACGGATT**AGACTTACCCCACAGCAAGAATC | GTCTGAGTTGAATTTATTTGAATTGCCTTT | AATTAGACTTACCCCACAGCAAGAAT |
| Oc29764_123 | 538828845 | T/C | **GAAGGTGACCAAGTTCATGCT**CAGACAAGAAACACGAAAGTCTCA | **GAAGGTCGGAGTCAACGGATT**CAGACAAGAAACACGAAAGTCTCG | GATTCTTTGCAGGAATAAGTGGCAGAT | CAGACAAGAAACACGAAAGTCTC |
| Oc29764_139 | 538828847 | A/C | **GAAGGTGACCAAGTTCATGCT**GAATAAGTGGCAGATGGTTTTGGTAAAT | **GAAGGTCGGAGTCAACGGATT**AATAAGTGGCAGATGGTTTTGGTAAAG | AGGCAATTCAAATAAATTCAACTCAGACAA | GAATAAGTGGCAGATGGTTTTGGTAAA |
| Oc08550_207 | 538828848 | T/G | **GAAGGTGACCAAGTTCATGCT**GACCCCTCTCTACCTAACCTA | **GAAGGTCGGAGTCAACGGATT**GACCCCTCTCTACCTAACCTC | TGTGACAAAGGTGAAGAATGGGTGAAT | GACCCCTCTCTACCTAACCT |
| Oc21511_102 | 538828850 | T/A | **GAAGGTGACCAAGTTCATGCT**CAGTTCTTGGTAGCGTGCCACTA | **GAAGGTCGGAGTCAACGGATT**CAGTTCTTGGTAGCGTGCCACTT | ATAAATACATAACCCACATTCTCTGAGCAA | CAGTTCTTGGTAGCGTGCCACT |
| Oc19209_110 | 538828851 | G/A | **GAAGGTGACCAAGTTCATGCT**GGGTGTTCAGGTGATCAACGC | **GAAGGTCGGAGTCAACGGATT**GGGGTGTTCAGGTGATCAACGT | GTTCATTCAACTACAGTGACGGCTTATAA | GGGTGTTCAGGTGATCAACG |
| Oc28044_197 | 538828853 | A/C | **GAAGGTGACCAAGTTCATGCT**ACCGAACTGACATTTGGAGAACTATAT | **GAAGGTCGGAGTCAACGGATT**CCGAACTGACATTTGGAGAACTATAG | AAAGGATGGCTATTGGCATTCTGA | ACCGAACTGACATTTGGAGAACTATA |
| Oc16834_320 | 538828855 | C/A | **GAAGGTGACCAAGTTCATGCT**TCATAAAGCATGATATACAGTTATAGATG | **GAAGGTCGGAGTCAACGGATT**CCTTCATAAAGCATGATATACAGTTATAGATT | ATTACATACAGCTTATAAAGCCTTCATAAA | TCATAAAGCATGATATACAGTTATAGAT |
| Oc16834_422 | 538828856 | T/C | **GAAGGTGACCAAGTTCATGCT**CCAAATGATCTAACAGTATGCACTGTA | **GAAGGTCGGAGTCAACGGATT**CAAATGATCTAACAGTATGCACTGTG | ATCTCAGCCTTGAATATTTCCAGTA | CCAAATGATCTAACAGTATGCACTGT |
| Oc19939_215 | 538828858 | A/C | **GAAGGTGACCAAGTTCATGCT**AGTGCGTGGACGTCGGCGT | **GAAGGTCGGAGTCAACGGATT**GTGCGTGGACGTCGGCGG | GTCCACCTGTCGACGATCACGAA | AGTGCGTGGACGTCGGCG |
| Oc08698_371 | 538828859 | C/T | **GAAGGTGACCAAGTTCATGCT**CCGAATACAGATAGGATTGATGGAG | **GAAGGTCGGAGTCAACGGATT**ACCGAATACAGATAGGATTGATGGAA | CTGATATGTCTGAGGGTTCTGAGTCTT | CCGAATACAGATAGGATTGATGGA |
| Oc13683_91 | 538828861 | G/A | **GAAGGTGACCAAGTTCATGCT**CCAGTGGGTTGTTAGTTAGAGGC | **GAAGGTCGGAGTCAACGGATT**CCAGTGGGTTGTTAGTTAGAGGT | TCAACAACCGATAACACACCACCCT | CCAGTGGGTTGTTAGTTAGAGG |
| Oc31038_260 | 538828863 | G/A | **GAAGGTGACCAAGTTCATGCT**TTCCCTCCGAGAGGCACTC | **GAAGGTCGGAGTCAACGGATT**CTTTCCCTCCGAGAGGCACTT | GTCAAGGCAGCAATTTATAGTTTGAGGTA | TTCCCTCCGAGAGGCACT |
| Oc19977_209 | 538828864 | A/G | **GAAGGTGACCAAGTTCATGCT**CATGGTAATTGTTCTCTGTAATAGCTATTTT | **GAAGGTCGGAGTCAACGGATT**ATGGTAATTGTTCTCTGTAATAGCTATTTC | CACCCTTCAAAAGATTAGAATCTTGGTAAT | CATGGTAATTGTTCTCTGTAATAGCTATTT |
| Oc16845_284 | 538828866 | T/C | **GAAGGTGACCAAGTTCATGCT**CAATCCCTTTAAAGCTGGAAATCATAG | **GAAGGTCGGAGTCAACGGATT**AACAATCCCTTTAAAGCTGGAAATCATAA | CATCTTTGTTGTTATATTGCAAACGGAGGT | CAATCCCTTTAAAGCTGGAAATCATA |
| Oc22435_202 | 538828867 | C/T | **GAAGGTGACCAAGTTCATGCT**GACTGTATGTGACTGTGTGAGTG | **GAAGGTCGGAGTCAACGGATT**GTGACTGTATGTGACTGTGTGAGTA | GCACCGCAAATGTTGCAATGAGCAA | GACTGTATGTGACTGTGTGAGT |
| Oc30238_165 | 538828869 | A/T | **GAAGGTGACCAAGTTCATGCT**GGACAAGCAAGTGGGCGTGCT | **GAAGGTCGGAGTCAACGGATT**GGACAAGCAAGTGGGCGTGCA | GTAGTTTCCAACAGGCCAATATTCTACAT | GGACAAGCAAGTGGGCGTGC |
| Oc23028_86 | 538828871 | C/T | **GAAGGTGACCAAGTTCATGCT**GGTGCGTGCGGAGCAATG | **GAAGGTCGGAGTCAACGGATT**CTGGTGCGTGCGGAGCAATA | TGTTTTGAGTCTGGTAGCGCAA | GGTGCGTGCGGAGCAAT |
| Oc13003_75 | 538828873 | T/A | **GAAGGTGACCAAGTTCATGCT**GAGTGAGAGGAGAACATGGAAACA | **GAAGGTCGGAGTCAACGGATT**GAGTGAGAGGAGAACATGGAAACT | AAAGTCAATAGTTAATGGACATGATGAGTA | GAGTGAGAGGAGAACATGGAAAC |
| Oc07934_362 | 538828874 | T/A | **GAAGGTGACCAAGTTCATGCT**CTCCCACGCGCTAACGTTAGA | **GAAGGTCGGAGTCAACGGATT**CTCCCACGCGCTAACGTTAGT | GCCATCAGCTGTTTTGTTAGCATGCAT | CTCCCACGCGCTAACGTTAG |
| Oc29335_82 | 538828876 | A/G | **GAAGGTGACCAAGTTCATGCT**TCATCTTCTGAGTTTCTAACCATTTCAAT | **GAAGGTCGGAGTCAACGGATT**CATCTTCTGAGTTTCTAACCATTTCAAC | TCATGAATGGTCAGACTTTATGAGACCA | TCATCTTCTGAGTTTCTAACCATTTCAA |
| Oc13003_178 | 538828878 | G/A | **GAAGGTGACCAAGTTCATGCT**ACAATCATGCAGGTGGATACGTTC | **GAAGGTCGGAGTCAACGGATT**GACAATCATGCAGGTGGATACGTTT | GGATGACGTAAAACCGTACAAATTCTGTT | ACAATCATGCAGGTGGATACGTT |
| Oc00809_502 | 538828879 | T/C | **GAAGGTGACCAAGTTCATGCT**GGAAACGCTGGCCTGAAGATCA | **GAAGGTCGGAGTCAACGGATT**GAAACGCTGGCCTGAAGATCG | GTGGTTAAACGCGGGTACTGTC | GGAAACGCTGGCCTGAAGATC |
| Oc04074_265 | 538828882 | T/C | **GAAGGTGACCAAGTTCATGCT**GCAGAGTTTATCAATTCAATTGTGGAAATATA | **GAAGGTCGGAGTCAACGGATT**CAGAGTTTATCAATTCAATTGTGGAAATATG | CATATTATTGAGGTGTCAAATCACCATGTA | GCAGAGTTTATCAATTCAATTGTGGAAATAT |
| Oc05679_298 | 538828883 | C/T | **GAAGGTGACCAAGTTCATGCT**CTTCTGGGTAACCAAATTCCG | **GAAGGTCGGAGTCAACGGATT**CCTCTTCTGGGTAACCAAATTCCA | TTCGGTAGTTGGTGGAGTTCGCAA | CTTCTGGGTAACCAAATTCC |
| Oc05742_114 | 538828885 | T/G | **GAAGGTGACCAAGTTCATGCT**ACTCGGGTGTACGTGGCTTTAAATA | **GAAGGTCGGAGTCAACGGATT**CGGGTGTACGTGGCTTTAAATC | TCTATCTATCCATCTTGTCTTGTTGGCAA | ACTCGGGTGTACGTGGCTTTAAAT |
| Oc07920_333 | 538828887 | G/T | **GAAGGTGACCAAGTTCATGCT**GATGAAATGTGATGTTAGCCTTCTAAC | **GAAGGTCGGAGTCAACGGATT**GAGATGAAATGTGATGTTAGCCTTCTAAA | TGGTCACTGACCGGGTAGAACTTT | GATGAAATGTGATGTTAGCCTTCTAA |
| Oc08698_95 | 538828888 | A/C | **GAAGGTGACCAAGTTCATGCT**TTACATTTAACATTTACATTTTAGTCATAGAT | **GAAGGTCGGAGTCAACGGATT**TACATTTAACATTTACATTTTAGTCATAGAG | TCAGTTGGTATTTCATGGTGCTTGCAA | TTACATTTAACATTTACATTTTAGTCATAGA |
| Oc08804_363 | 538828890 | A/G | **GAAGGTGACCAAGTTCATGCT**GGTCTGTTATGATGCTCAAGTCTGT | **GAAGGTCGGAGTCAACGGATT**GTCTGTTATGATGCTCAAGTCTGC | CAAGCTCTGGGGAGAAGTGCTGAA | GGTCTGTTATGATGCTCAAGTCTG |
| Oc08992_271 | 538828892 | G/A | **GAAGGTGACCAAGTTCATGCT**CCGGATTCGACCATATTAATGACC | **GAAGGTCGGAGTCAACGGATT**TCCGGATTCGACCATATTAATGACT | GCGTACGAGCTGGTGCTTACCAT | CCGGATTCGACCATATTAATGAC |
| Oc09037_130 | 538828894 | A/G | **GAAGGTGACCAAGTTCATGCT**GCAGGAGCGCTTAGTATTTCAACAAT | **GAAGGTCGGAGTCAACGGATT**CAGGAGCGCTTAGTATTTCAACAAC | AGCCTAAATGCTAAATGCATAGGCATGTT | GCAGGAGCGCTTAGTATTTCAACAA |
| Oc11463_340 | 538828895 | C/T | **GAAGGTGACCAAGTTCATGCT**ACGATTGTTGAAATACTATAAAAGACTGAG | **GAAGGTCGGAGTCAACGGATT**AAACGATTGTTGAAATACTATAAAAGACTGAA | GCAAGATCAGCCATCGCTCATTCAA | ACGATTGTTGAAATACTATAAAAGACTGA |
| Oc12003_252 | 538828897 | G/A | **GAAGGTGACCAAGTTCATGCT**AAATAACACCTGGGCCAGTGGC | **GAAGGTCGGAGTCAACGGATT**AAATAACACCTGGGCCAGTGGT | AAAACATTTGGGTAGTTTGCCCCAACAA | AAATAACACCTGGGCCAGTGG |
| Oc12653_72 | 538828899 | A/C | **GAAGGTGACCAAGTTCATGCT**AAGTTTTGCAACAGAATCGGCTTTAATT | **GAAGGTCGGAGTCAACGGATT**AGTTTTGCAACAGAATCGGCTTTAATG | GGGCTTGATTGGTAGCGTTAACGTT | AAGTTTTGCAACAGAATCGGCTTTAAT |
| Oc12789_349 | 538828900 | A/G | **GAAGGTGACCAAGTTCATGCT**AGCTTTTTACACCTGACAGCTAAAGT | **GAAGGTCGGAGTCAACGGATT**GCTTTTTACACCTGACAGCTAAAGC | GAGCATTATCCTCTGTGATTTAATAGGTTA | AGCTTTTTACACCTGACAGCTAAAG |
| Oc14458_107 | 538828902 | A/G | **GAAGGTGACCAAGTTCATGCT**ATATTGTTGTCTTGTTACAGCACCTT | **GAAGGTCGGAGTCAACGGATT**ATATTGTTGTCTTGTTACAGCACCTC | CACTACCATTGATGAGATAAGACTGTCAT | ATATTGTTGTCTTGTTACAGCACCT |
| Oc15163_278 | 538828904 | A/G | **GAAGGTGACCAAGTTCATGCT**GTCAAATCCTCGCAATGTTATTGCAT | **GAAGGTCGGAGTCAACGGATT**GTCAAATCCTCGCAATGTTATTGCAC | GCCCTCTTATTGTTCTGTGATAAGAGTAT | GTCAAATCCTCGCAATGTTATTGCA |
| Oc15873_229 | 538828905 | T/C | **GAAGGTGACCAAGTTCATGCT**ACGTTCATATGTTCACACGGTCAATA | **GAAGGTCGGAGTCAACGGATT**CGTTCATATGTTCACACGGTCAATG | GCAGCACTGTGGAGGAGTAACATAT | ACGTTCATATGTTCACACGGTCAAT |
| Oc17156_195 | 538828907 | T/A | **GAAGGTGACCAAGTTCATGCT**AAGTCAAACAAAACAATATCTTTATTATCAATA | **GAAGGTCGGAGTCAACGGATT**AAGTCAAACAAAACAATATCTTTATTATCAATT | GGATGCTTATAGGGAAGGACATTCAATAA | AAGTCAAACAAAACAATATCTTTATTATCAAT |
| Oc17280_103 | 538828909 | C/G | **GAAGGTGACCAAGTTCATGCT**ACTTTTTCATTACAGAGACATAAACACAG | **GAAGGTCGGAGTCAACGGATT**ACTTTTTCATTACAGAGACATAAACACAC | GTAGAAAGTAAACATCCGCACCTCGA | ACTTTTTCATTACAGAGACATAAACACA |
| Oc17530_333 | 538828910 | C/T | **GAAGGTGACCAAGTTCATGCT**GCATGGAATATTTTACTTTGCAGTGAG | **GAAGGTCGGAGTCAACGGATT**ATGCATGGAATATTTTACTTTGCAGTGAA | CACCGGACACCATGTCCAGCTT | GCATGGAATATTTTACTTTGCAGTGA |
| Oc17878_324 | 538828912 | A/G | **GAAGGTGACCAAGTTCATGCT**ATTATACTCTATATGCTGTCTATACATTTAGTT | **GAAGGTCGGAGTCAACGGATT**ATACTCTATATGCTGTCTATACATTTAGTC | CCTGTGGAAAGCTTTCGACACCTT | ATTATACTCTATATGCTGTCTATACATTTAGT |
| Oc18640_82 | 538828914 | C/A | **GAAGGTGACCAAGTTCATGCT**ACACTTGCATTCTTGGGCTATAGG | **GAAGGTCGGAGTCAACGGATT**CACACTTGCATTCTTGGGCTATAGT | CGTTTGAGAACCCCAACAATAGTACTTAA | ACACTTGCATTCTTGGGCTATAG |
| Oc19628_318 | 538828915 | T/C | **GAAGGTGACCAAGTTCATGCT**ACAATTGAGTAAAACTTGTAACGTATTAATATA | **GAAGGTCGGAGTCAACGGATT**ACAATTGAGTAAAACTTGTAACGTATTAATATG | TTGTTAATAGCTGAGATTTTAATGGCCGTA | ACAATTGAGTAAAACTTGTAACGTATTAATAT |
| Oc20655_157 | 538828917 | A/G | **GAAGGTGACCAAGTTCATGCT**GATAATTTATAACACTTTTACAGCCATTCAAAT | **GAAGGTCGGAGTCAACGGATT**ATAATTTATAACACTTTTACAGCCATTCAAAC | TGAACAAGGCATTATACACCCAGGATTT | GATAATTTATAACACTTTTACAGCCATTCAAA |
| Oc22333_123 | 538828919 | T/G | **GAAGGTGACCAAGTTCATGCT**CGTCCTCCCCTCAGCAGCA | **GAAGGTCGGAGTCAACGGATT**GTCCTCCCCTCAGCAGCC | GCCGTTTATGGAGAGAGATCGTGA | CGTCCTCCCCTCAGCAGC |
| Oc22338_383 | 538828920 | A/C | **GAAGGTGACCAAGTTCATGCT**AGCAGCAGACCAAAGCAGAGGT | **GAAGGTCGGAGTCAACGGATT**GCAGCAGACCAAAGCAGAGGG | ACCACTGTTTAAGCATAAAGCCAGCATTT | AGCAGCAGACCAAAGCAGAGG |
| Oc22462_375 | 538960845 | T/C | **GAAGGTGACCAAGTTCATGCT**CCTTTTTACATTAACAGATGTCACAAAGTA | **GAAGGTCGGAGTCAACGGATT**CTTTTTACATTAACAGATGTCACAAAGTG | ACATCTGCATTGCTTGCTGTTTACAGTTT | CCTTTTTACATTAACAGATGTCACAAAGT |
| Oc22599_343 | 538828922 | A/G | **GAAGGTGACCAAGTTCATGCT**AATATGTTGTGATGCTTGTCTTACCAAAAT | **GAAGGTCGGAGTCAACGGATT**ATGTTGTGATGCTTGTCTTACCAAAAC | CCATGGCACACTAACGACTTCAGTT | AATATGTTGTGATGCTTGTCTTACCAAAA |
| Oc22915_171 | 538828923 | T/A | **GAAGGTGACCAAGTTCATGCT**ACAAGGTAGTGGAAAGCAGTGGA | **GAAGGTCGGAGTCAACGGATT**ACAAGGTAGTGGAAAGCAGTGGT | TGTGTAAGATAAGGAGGAGTATAGGGAA | ACAAGGTAGTGGAAAGCAGTGG |
| Oc23782_101 | 538828925 | A/C | **GAAGGTGACCAAGTTCATGCT**CCGTTATCCTTTTGTACAAAGCACAT | **GAAGGTCGGAGTCAACGGATT**CCGTTATCCTTTTGTACAAAGCACAG | CTCCCAAATTTAACAAACTGTGCCAGTAA | CCGTTATCCTTTTGTACAAAGCACA |
| Oc24605_203 | 538828927 | G/A | **GAAGGTGACCAAGTTCATGCT**TTAACATTAACATTAACATTTCTATTTGCTTC | **GAAGGTCGGAGTCAACGGATT**TAACATTAACATTAACATTTCTATTTGCTTT | AGGAGTGCTTCATTAAGACTCTTGACATA | TTAACATTAACATTAACATTTCTATTTGCTT |
| Oc25006_99 | 538828928 | A/C | **GAAGGTGACCAAGTTCATGCT**ACACTTCAAGAATGTTTAATGTTACGACT | **GAAGGTCGGAGTCAACGGATT**CACTTCAAGAATGTTTAATGTTACGACG | CCTTCGGTCGGAGTTGTGACAAAAT | ACACTTCAAGAATGTTTAATGTTACGAC |
| Oc26415_290 | 538828930 | A/G | **GAAGGTGACCAAGTTCATGCT**CGTTCAGTTCAGCTGGAAACTT | **GAAGGTCGGAGTCAACGGATT**CGTTCAGTTCAGCTGGAAACTC | GAGGAGTAAATGTTTGGCGACCAGAA | CGTTCAGTTCAGCTGGAAACT |
| Oc27198_223 | 538828931 | T/C | **GAAGGTGACCAAGTTCATGCT**AGAACTCAGGTGATGAAGATGTAAATATTA | **GAAGGTCGGAGTCAACGGATT**GAACTCAGGTGATGAAGATGTAAATATTG | AGATTCAGTGATGAGATTTCCACCGTTT | AGAACTCAGGTGATGAAGATGTAAATATT |
| Oc27616_127 | 538828933 | T/C | **GAAGGTGACCAAGTTCATGCT**GTGACTCCCTGTCAAAGTATATGTTATAA | **GAAGGTCGGAGTCAACGGATT**GACTCCCTGTCAAAGTATATGTTATAG | CATATTCACAGTCTTGCCAGGCTGTT | GTGACTCCCTGTCAAAGTATATGTTATA |
| Oc29155_193 | 538828935 | A/G | **GAAGGTGACCAAGTTCATGCT**GCATTGGTTTCCATCTTTGTTACATCT | **GAAGGTCGGAGTCAACGGATT**CATTGGTTTCCATCTTTGTTACATCC | ACCCGTCTTCCACATTGTGCTCTT | GCATTGGTTTCCATCTTTGTTACATC |
| Oc30028_263 | 538828936 | A/G | **GAAGGTGACCAAGTTCATGCT**TAAATAAATAATGATCTTCAGAAATTACTTTA | **GAAGGTCGGAGTCAACGGATT**AAATAAATAATGATCTTCAGAAATTACTTTG | ACCACAAACCTACTCAAAGTTTTCACCAT | TAAATAAATAATGATCTTCAGAAATTACTTT |
| Oc31757_279 | 538828938 | C/T | **GAAGGTGACCAAGTTCATGCT**ATGACCTCAAGAAATCCCAGCTTC | **GAAGGTCGGAGTCAACGGATT**GATGACCTCAAGAAATCCCAGCTTT | TTAAAGGCGGTCTTCCTCATCTTTTCTTT | ATGACCTCAAGAAATCCCAGCTT |
| Oc32144_118 | 538828940 | A/G | **GAAGGTGACCAAGTTCATGCT**AAGCGTGGTCATTAGGCCTATAGT | **GAAGGTCGGAGTCAACGGATT**GCGTGGTCATTAGGCCTATAGC | GTGCTATTTGCATGTAAAAATGAACGCCAT | AAGCGTGGTCATTAGGCCTATAG |
| Oc36061_187 | 538828942 | T/A | **GAAGGTGACCAAGTTCATGCT**AGAATCCCAGATACCCTCCATGTA | **GAAGGTCGGAGTCAACGGATT**AGAATCCCAGATACCCTCCATGTT | CATCCTTGTCCATCAGAGGACATCT | AGAATCCCAGATACCCTCCATGT |
