# Supplementary material for: Single nucleotide polymorphism discovery in cutthroat trout subspecies using genome reduction, barcoding, and 454 pyro-sequencing
Source: BMC Genomics. 2012 Dec 23;13:724. doi: 10.1186/1471-2164-13-724 (PMC3549761; doi:10.1186/1471-2164-13-724)
Supplement: Additional file 2 — Characterization of each SNP locus, Major and minor alleles for all 125 SNP loci, along with the proportions of individuals within each a priori designated subspecies that carry the minor allele, as well as minor allele frequencies for each SNP locus are listed herein. [file 1471-2164-13-724-S2.docx]

**Additional File 2**: Trout lineages that carry each SNP that was characterized. Major and minor alleles for all 125 SNP loci, along with the proportions of individuals within each *a priori* designated subspecies that carry the minor allele and the minor allele frequencies of each SNP locus are listed. Asterisks denote instances when at least one individual carrying the minor allele was determined to carry alleles from multiple cutthroat subspecies in our Structure analyses. Abbreviations for subspecies are as follows: Bear River (BR), Bonneville (BON), Coastal (COA), Colorado River (CR), Greenback (GR), Lahontan Basin (LAH), Rio Grande (RG), Westslope (WS), Yellowstone (YS), and rainbow trout (RBT).

|  |  |  | Proportions of Individuals Carrying Minor Alleles by Subspecies | | | | | | | | | |  |
| --- | --- | --- | --- | --- | --- | --- | --- | --- | --- | --- | --- | --- | --- |
| SNP ID | Major Allele | Minor Allele | BR | BON | COA | CR | GR | LAH | RG | WS | YS | RBT | Minor Allele Frequency |
| Oc18318_297 | G | T | 0.05* | – | 1.00* | – | 0.05* | – | – | 1.00 | – | – | 0.22043 |
| Oc02855_246 | C | T | 0.05* | 0.05* | 0.05* | – | 0.05* | 0.90 | – | – | – | – | 0.11828 |
| Oc00719_477 | T | G | 0.05* | – | – | – | 0.05* | – | – | 0.80 | – | – | 0.107527 |
| Oc01000_503 | A | C | 0.10* | 0.10 | 0.90* | – | – | 0.10* | – | – | – | 1.00 | 0.182796 |
| Oc04520_300 | A | G | 0.90* | 0.10* | – | 0.15* | 0.05* | – | 0.20* | 1.00 | 0.90* | – | 0.39759 |
| Oc07619_342 | T | A | 0.05* | 0.15* | 1.00* | – | 0.05 | 0.05 | 0.10 | 1.00 | – | – | 0.282353 |
| Oc02334_453 | A | G | 0.10* | – | – | 0.70* | 0.60 | – | – | – | – | – | 0.152174 |
| Oc07137_365 | C | T | 0.70* | 0.95* | – | 0.05* | 0.05* | – | 0.10* | – | 0.90* | – | 0.292553 |
| Oc11603_128 | C | A | – | – | – | 0.10* | 0.10* | – | 1.00* | – | – | – | 0.12766 |
| Oc01121_395 | T | C | – | – | 1.00* | – | – | 1.00* | 0.25* | 1.00 | – | 1.00 | 0.403226 |
| Oc01727_205 | T | C | – | – | 1.00* | – | – | 0.10* | – | 1.00 | – | 1.00 | 0.27957 |
| Oc04584_342 | T | G | 0.65* | – | 0.10* | – | 0.05* | – | 0.05* | – | 0.65* | – | 0.163043 |
| Oc00002_837 | A | G | 0.05* | 0.15* | 0.95* | – | 0.05* | 1.00* | 0.10* | 0.95 | – | 1.00 | 0.403226 |
| Oc07779_247 | T | G | 0.10* | 0.15* | 0.90* | – | 0.05* | 0.95* | 0.10 | – | – | 1.00 | 0.292553 |
| Oc22333_295 | T | A | 0.05* | – | 0.95* | 0.05* | 0.05* | 1.00* | – | – | – | 0.80 | 0.268817 |
| Oc06689_176 | T | A | 0.10* | 0.15* | 0.10* | 0.50* | 0.10* | 0.05 | 0.10 | – | – | – | 0.126437 |
| Oc04368_474 | G | C | 0.35* | 0.40* | 0.25* | 0.55* | 0.70* | – | 0.80* | 0.35 | 0.30* | 0.40 | 0.419355 |
| Oc00162_353 | C | A | – | – | – | – | – | – | – | 1.00 | – | – | 0.107527 |
| Oc06563_94 | G | A | 0.30* | 0.05* | – | 0.10* | 0.05 | – | 0.30* | 0.90 | 0.45* | 0.10 | 0.241758 |
| Oc01301_225 | C | A | 0.40* | 0.40* | 0.35* | 0.95* | 0.95* | – | 1.00* | – | 0.15* | 0.10 | 0.452128 |
| Oc01849_333 | T | C | 0.95* | 1.00* | – | 0.75* | 0.25* | – | 0.25* | – | 0.90* | – | 0.44086 |
| Oc04318_267 | T | A | 0.05* | 0.10* | 0.05* | 0.50* | 0.25* | – | – | – | 0.05* | – | 0.107527 |
| Oc04332_250 | G | A | 0.70* | 0.15* | – | – | 0.10* | – | – | – | 0.40* | – | 0.145161 |
| Oc13395_65 | T | C | 0.05* | 0.10* | 0.10* | 0.55* | 0.70* | 0.05 | 0.75* | – | – | – | 0.25 |
| Oc02233_468 | T | A | 0.10* | – | 0.10* | 0.70* | 0.55* | – | 0.35* | – | 0.10* | 0.10 | 0.209677 |
| Oc04162_306 | T | C | – | – | – | – | – | – | – | 1.00 | – | – | 0.113636 |
| Oc03207_330 | A | T | 0.25* | – | – | – | 0.05* | – | – | – | – | – | 0.037975 |
| Oc02284_320 | T | C | – | – | – | – | – | 0.90 | – | – | – | – | 0.096774 |
| Oc02956_408 | G | A | 0.05* | 0.10* | 0.10* | – | 0.05* | 0.90 | 0.10 | – | – | – | 0.139785 |
| Oc09421_125 | A | T | 0.10* | 0.20* | 0.10* | 0.75* | 0.25* | 0.05 | 0.75* | – | – | – | 0.278481 |
| Oc04740_310 | G | A | 0.05* | – | – | 0.05* | 0.05* | – | 0.60* | – | – | – | 0.083333 |
| Oc09353_200 | G | A | 0.80* | 0.10* | 0.10* | 0.10* | 0.15* | – | 0.20* | 0.50 | 0.90* | 0.10 | 0.311828 |
| Oc00078_436 | C | A | – | – | 0.90* | – | 0.15* | 0.85* | 0.50 | 1.00 | – | 1.00 | 0.419355 |
| Oc02899_311 | A | C | 0.50* | 0.55* | 0.50* | 0.50* | 0.50* | 0.15* | 0.55* | 0.30 | 0.45* | 0.50 | 0.456989 |
| Oc01496_132 | C | T | 1.00* | 1.00* | – | 0.05* | 0.10* | – | 0.30* | – | 1.00* | – | 0.363158 |
| Oc15008_273 | T | A | – | – | 1.00* | – | – | 0.90 | – | 1.00 | – | 1.00 | 0.32967 |
| Oc22659_144 | A | G | 1.00* | 0.50* | – | 0.20* | 0.10* | – | 0.10* | – | 1.00* | – | 0.305263 |
| Oc27906_245 | T | A | – | 0.20 | – | – | 0.05* | – | – | – | – | – | 0.026316 |
| Oc23835_105 | C | A | 0.20* | 0.95* | – | 0.15* | 0.10* | – | – | – | – | – | 0.147368 |
| Oc12661_311 | A | G | 0.30* | 0.90* | – | 0.20* | – | – | 1.00* | – | 0.30* | – | 0.287234 |
| Oc25168_210 | G | T | – | – | 0.30* | – | – | – | – | – | – | – | 0.031579 |
| Oc15656_263 | G | T | 0.50* | – | – | – | 0.10* | 0.90 | 1.00* | – | 1.00* | – | 0.368421 |
| Oc17530_158 | T | C | – | – | 0.70* | – | – | – | – | – | – | – | 0.073684 |
| Oc12934_230 | T | A | – | – | – | 0.75* | 0.30* | – | 0.80* | – | – | – | 0.194737 |
| Oc11988_119 | G | A | – | – | – | 0.05* | – | 0.10* | – | – | – | 1.00 | 0.068421 |
| Oc24645_268 | C | G | – | 0.05* | – | 0.75* | 0.05 | – | – | – | – | – | 0.089474 |
| Oc25786_318 | A | T | – | – | – | 0.70* | 0.30* | – | – | – | – | – | 0.106383 |
| Oc17643_137 | G | A | – | – | – | 0.80* | 0.20* | – | – | – | – | – | 0.105263 |
| Oc06689_176 | T | A | – | – | – | 0.50* | 0.10* | – | – | – | – | – | 0.065934 |
| Oc07334_176 | C | A | – | – | 1.00* | – | 0.05* | 0.95* | – | 1.00 | – | 1.00 | 0.368421 |
| Oc00345_62 | C | A | – | 0.10* | – | 0.20 | 0.40* | – | – | – | – | – | 0.073684 |
| Oc32430_172 | C | T | – | – | – | – | 0.20* | – | 0.40* | – | – | – | 0.064516 |
| Oc35801_240 | C | T | – | – | 1.00* | 0.20* | 0.90* | 0.10* | 0.20 | 1.00 | – | 1.00 | 0.410526 |
| Oc06387_477 | C | T | 0.10* | 0.50* | – | – | 0.10* | – | – | – | – | – | 0.073684 |
| Oc15945_33 | A | G | – | – | – | – | – | 0.90 | – | – | – | – | 0.094737 |
| Oc06930_61 | G | T | 0.05* | – | – | – | – | 0.95* | – | – | 0.05* | 0.40 | 0.131579 |
| Oc20487_212 | C | T | – | – | – | – | – | 0.80 | – | – | – | – | 0.086022 |
| Oc21247_311 | G | A | – | – | – | – | – | 0.50 | – | – | – | – | 0.052632 |
| Oc25620_232 | A | G | – | – | – | – | – | 0.90 | – | – | – | – | 0.094737 |
| Oc15641_178 | A | C | – | – | – | – | – | 0.60 | – | – | – | – | 0.063158 |
| Oc13704_250 | C | T | – | – | – | – | – | 0.90 | – | – | – | – | 0.410526 |
| Oc38332_102 | C | A | – | – | – | – | – | 0.90 | – | – | – | – | 0.094737 |
| Oc21532_151 | A | C | 0.20* | 0.70* | – | 0.20* | – | – | 0.80* | – | – | – | 0.2 |
| Oc29764_214 | C | T | – | – | – | – | – | 0.40 | 1.00* | – | – | – | 0.171429 |
| Oc29764_66 | A | C | 0.90* | 0.20* | 0.10* | 0.85* | 0.75* | 0.05* | 0.20* | – | 1.00 | – | 0.426316 |
| Oc29764_123 | G | A | 0.40* | 0.10* | – | 0.65* | 0.25* | – | 0.15* | – | 0.65* | – | 0.234043 |
| Oc29764_139 | A | C | – | – | – | – | – | 1.00* | 1.00* | – | – | – | 0.215054 |
| Oc08550_207 | C | A | – | – | – | – | – | – | 0.90* | – | – | – | 0.094737 |
| Oc21511_102 | A | T | – | – | – | – | – | – | 0.75* | – | 0.05* | – | 0.085106 |
| Oc19209_110 | T | C | – | – | – | 0.10 | 0.65* | – | 0.90* | 0.05 | – | – | 0.180851 |
| Oc28044_197 | A | C | – | – | 0.55* | – | – | 0.35* | 0.05 | 1.00 | – | 1.00 | 0.272222 |
| Oc16834_320 | A | C | 0.50* | 0.50* | 0.50* | 0.50* | 0.50* | 0.55* | 0.50* | – | 0.50* | 0.95 | 0.478947 |
| Oc16834_422 | G | A | – | – | – | – | – | – | – | 1.00 | – | – | 0.105263 |
| Oc19939_215 | C | A | – | – | – | – | – | 0.10* | – | 1.00 | – | 1.00 | 0.168421 |
| Oc08698_371 | C | T | – | – | – | – | – | – | – | 1.00 | – | – | 0.106383 |
| Oc13683_91 | T | C | – | – | 1.00* | – | – | 0.10* | – | 1.00 | – | 1.00 | 0.273684 |
| Oc31038_260 | C | T | – | – | – | – | – | – | – | 1.00 | – | – | 0.106383 |
| Oc19977_209 | A | G | 0.20* | – | – | – | – | – | – | 1.00 | – | – | 0.126316 |
| Oc16845_284 | T | C | – | – | 1.00* | – | – | 0.10* | – | 1.00 | – | 1.00 | 0.273684 |
| Oc22435_202 | C | T | – | – | 0.20* | – | – | – | – | 1.00 | – | – | 0.126316 |
| Oc30238_165 | T | A | 0.30 | – | – | – | – | – | – | – | 0.60* | – | 0.094737 |
| Oc23028_86 | T | C | – | – | – | 0.10* | – | – | – | – | 0.70* | – | 0.094118 |
| Oc13003_75 | T | A | 0.45* | 0.10* | – | 0.05* | 0.10* | – | 0.15* | – | 0.85* | – | 0.180851 |
| Oc07934_362 | T | A | – | – | – | – | – | – | – | – | 0.20* | – | 0.021053 |
| Oc29335_82 | G | A | 0.60* | 0.05* | 0.70* | 0.10* | 0.10* | – | 0.15* | – | 0.80* | – | 0.263158 |
| Oc13003_178 | C | T | 0.20* | 0.10* | – | 0.05* | 0.10* | – | 0.15* | – | 0.70* | – | 0.144444 |
| Oc00809_502 | A | G | – | – | – | 0.90* | 0.40* | – | – | – | – | – | 0.141304 |
| Oc04074_265 | G | A | 0.22* | 0.55* | – | 0.05* | 0.05* | 0.20 | 0.10 | 0.25 | 0.30* | – | 0.198864 |
| Oc05679_298 | T | C | – | – | – | – | – | – | – | – | 0.20* | – | 0.021277 |
| Oc05742_114 | C | A | – | 0.05* | – | 0.75* | 0.05 | – | – | – | – | – | 0.090426 |
| Oc07920_333 | T | G | – | – | – | 0.40* | 0.90* | – | – | – | – | – | 0.120879 |
| Oc08698_95 | A | C | – | – | 0.30* | – | – | – | – | 0.20 | 0.15* | – | 0.072222 |
| Oc08804_363 | A | G | 0.40* | 0.05* | 0.50* | 0.50* | 0.50* | 0.55* | 0.60* | 0.50 | 0.50* | 0.50 | 0.456522 |
| Oc08992_271 | C | T | 0.80* | – | 0.10* | – | – | – | – | – | 0.56* | – | 0.153846 |
| Oc09037_130 | G | A | 0.40* | 0.05* | 0.50* | 0.55* | 0.50* | 0.50 | 0.65* | 0.50 | 0.55* | 0.75 | 0.483696 |
| Oc11463_340 | C | T | – | – | – | – | 0.20* | – | 0.85* | – | – | – | 0.116667 |
| Oc12003_252 | C | T | 0.55* | 0.50* | 0.50* | 0.45* | 0.50* | 0.40* | 0.35* | 0.50 | 0.45* | 0.25 | 0.461957 |
| Oc12653_72 | C | A | 0.44* | 0.05 | – | 0.10* | 0.05 | – | 0.05* | – | 0.40* | – | 0.115385 |
| Oc12789_349 | G | A | 0.10* | 0.50* | 0.50* | 0.50* | 0.50* | 0.50* | 0.50* | – | – | 0.50 | 0.390244 |
| Oc14458_107 | G | A | 0.20* | – | – | – | – | – | – | 0.05* | – | – | 0.027174 |
| Oc15163_278 | A | G | 0.55* | 0.55* | 0.15 | 0.55* | 0.50* | 0.55* | 0.65* | 0.50 | 0.50 | – | 0.489011 |
| Oc15873_229 | G | A | 0.30* | 0.95* | – | – | 0.10* | – | – | – | 0.05* | – | 0.147368 |
| Oc17156_195 | T | A | – | 0.05* | – | 0.70* | 0.20* | – | – | – | – | – | 0.102151 |
| Oc17280_103 | C | G | – | – | – | 0.50* | 0.20* | – | 0.05* | – | – | – | 0.079787 |
| Oc17530_333 | C | T | 0.25* | 0.10* | 0.70* | 0.05* | – | 0.10* | – | 1.00 | 0.65* | 1.00 | 0.335165 |
| Oc17878_324 | G | A | 0.05* | 0.05 | 0.45* | – | – | 0.50* | 0.10* | 0.20 | – | 0.40 | 0.173913 |
| Oc18640_82 | T | G | – | 0.25 | – | 0.05* | 0.15* | – | – | – | – | – | 0.048913 |
| Oc19628_318 | A | G | 0.10* | 0.05* | – | 0.60* | 0.35* | – | – | – | – | – | 0.115789 |
| Oc20655_157 | A | G | – | – | – | 0.70* | 0.15* | – | – | – | – | – | 0.089474 |
| Oc22333_123 | C | A | 0.60* | 0.30* | 0.10 | – | 0.10* | – | – | – | 0.05* | – | 0.125 |
| Oc22338_383 | C | A | 0.20 | – | 0.20* | 0.22* | – | 0.20 | 0.30* | 0.10 | – | 0.50 | 0.144444 |
| Oc22462_375 | G | A | 0.15* | 0.05 | 0.20* | 0.10 | – | 0.15 | 0.30* | 0.10 | 0.10* | – | 0.129213 |
| Oc22599_343 | A | G | – | – | – | 0.25* | 0.05 | – | – | – | – | – | 0.033708 |
| Oc22915_171 | A | T | – | – | – | – | 0.15* | – | 0.95* | – | – | – | 0.119565 |
| Oc23782_101 | C | A | 0.15* | 0.05* | – | – | – | – | 0.20* | – | 0.75* | – | 0.121053 |
| Oc24605_203 | C | T | – | – | – | – | – | – | 0.90* | – | – | – | 0.095745 |
| Oc25006_99 | C | A | 0.45* | 0.05 | – | – | – | – | – | – | 0.06* | – | 0.06044 |
| Oc26415_290 | G | A | 0.30* | 0.10* | 0.65* | 0.45* | 0.40* | 0.70* | 0.55* | 0.70 | 0.35* | 0.50 | 0.467391 |
| Oc27198_223 | G | A | 0.60* | 0.72* | 0.05* | 0.40* | – | 0.70 | 0.70* | 0.70 | 0.65* | 0.40 | 0.494681 |
| Oc27616_127 | G | A | 0.65* | 0.10* | – | 0.15* | – | – | 0.05* | – | 0.40* | – | 0.143617 |
| Oc29155_193 | G | A | 1.00* | – | – | 0.15* | 0.05 | – | 0.25* | – | 1.00* | – | 0.257895 |
| Oc30028_263 | G | A | 0.05* | – | 1.00* | – | – | 0.90 | – | – | – | 0.10 | 0.213483 |
| Oc31757_279 | C | T | 0.25* | – | – | 0.05* | – | – | 0.07* | – | 1.00* | – | 0.138889 |
| Oc32144_118 | G | A | 0.45* | 0.05* | 0.50* | 0.50* | 0.45* | 0.50 | 0.50* | 0.50 | 0.55* | 0.50 | 0.445652 |
| Oc36061_187 | T | A | 0.05* | – | 0.05 | 0.60* | 0.35* | – | – | 0.05 | – | – | 0.117021 |
